# A corticostriatal pathway mediating self-efficacy enhancement

**DOI:** 10.1038/s44184-022-00006-7

**Published:** 2022-07-08

**Authors:** Ofir Shany, Guy Gurevitch, Gadi Gilam, Netta Dunsky, Shira Reznik Balter, Ayam Greental, Noa Nutkevitch, Eran Eldar, Talma Hendler

**Affiliations:** 1 School of Psychological Sciences, Tel-Aviv University, Tel-Aviv, Israel; 2 Sagol Brain Institute, Tel-Aviv Sourasky Medical Center, Tel-Aviv, Israel; 3 Sackler School of Medicine, Tel-Aviv University, Tel-Aviv, Israel; 4 The Institute of Biomedical and Oral Research, Faculty of Dental Medicine, Hebrew University of Jerusalem, Jerusalem, Israel; 5 Sagol School of Neuroscience, Tel-Aviv University, Tel-Aviv, Israel; 6 Psychology and Cognitive Sciences Departments, Hebrew University of Jerusalem, Jerusalem, Israel

**Keywords:** Personality, Striatum, Reward, Human behaviour

## Abstract

Forming positive beliefs about one’s ability to perform challenging tasks, often termed self-efficacy, is fundamental to motivation and emotional well-being. Self-efficacy crucially depends on positive social feedback, yet people differ in the degree to which they integrate such feedback into self-beliefs (i.e., positive bias). While diminished positive bias of this sort is linked to mood and anxiety, the neural processes by which positive feedback on public performance enhances self-efficacy remain unclear. To address this, we conducted a behavioral and fMRI study wherein participants delivered a public speech and received fictitious positive and neutral feedback on their performance in the MRI scanner. Before and after receiving feedback, participants evaluated their actual and expected performance. We found that reduced positive bias in updating self-efficacy based on positive social feedback associated with a psychopathological dimension reflecting symptoms of anxiety, depression, and low self-esteem. Analysis of brain encoding of social feedback showed that a positive self-efficacy update bias associated with a stronger reward-related response in the ventral striatum (VS) and stronger coupling of the VS with a temporoparietal region involved in self-processing. Together, our findings demarcate a corticostriatal circuit that promotes positive bias in self-efficacy updating based on social feedback, and highlight the centrality of such bias to emotional well-being.

## Introduction

People are often required to perform challenging tasks in order to achieve desired goals. Perceiving task performance as successful can strengthen individuals’ beliefs about their ability to succeed in similar tasks in the future, which are known as self-efficacy^1^. Positive beliefs about abilities are crucial for a person’s decision to engage in a task or to avoid it, and are respectively linked to professional success and emotional well-being^2–4^. People cultivate such beliefs primarily as a result of past success^5^, which is estimated to a substantial degree based on social feedback^6,7^. Social feedback is particularly important in stressful situations in which we are uncertain about our performance and are exposed to feedback from other people about it. Hence, positive social feedback constitutes a powerful success indicator that can reinforce beliefs regarding one’s ability to achieve the desired outcomes^2^. Here we aim to characterize neuropsychological processes that contribute to individual differences in the effect of positive social feedback on updating self-beliefs concerning a challenging social performance. Specifically, we examine how participants integrate feedback into re-assessments of their performance in a previous public speaking task; and particularly into assessments about their expected performance in a similar upcoming task (i.e., self-efficacy).

Wide individual variability exists in the manner by which people process information about their performance. Most individuals strive for self-enhancement and improvement^8^. Respectively, people tend to expect that they will perform well and receive positive feedback in various contexts, and this tendency is increased among individuals with higher trait levels of self-efficacy and self-esteem^2,7,9–12^. Moreover, positive feedback is often accepted unquestioningly and integrated into evaluations of one’s performance and attributes, relative to undesirable feedback^4,13^. However, individuals with symptoms of mood and anxiety disorders commonly display diminished “positive bias” of this sort, and tend to evaluate their performance negatively even in the face of positive feedback. Thus, instead of imagining success, they focus on personal deficiencies and possible negative outcomes during and in-between task performances^9,11,14,15^. Such individuals are less likely to integrate positive feedback into evaluations of a past performance relative to less desirable feedbacks^4,16,17^, and also assign greater weight to negative versus positive feedback while forming beliefs about their ability to master a novel task (i.e., self-efficacy)^18^. This diminished positive bias is particularly dominant among people with high social anxiety, who have a poor sense of self-efficacy regarding socio-evaluative situations^15,19^. Diminished positive bias may thus impede individuals from cultivating a positive sense of self-efficacy. In turn, this could strengthen avoidance tendencies from social performative contexts with which the individual is struggling, and aggravate symptoms of anxiety and depression.

An effective usage of positive feedback for updating performance estimates involves registering the reward value of the feedback and attributing it to oneself. This process is thought to rely on the mesolimbic reward circuit—especially the ventral striatum (VS), which generates positive affective responses and guides reward learning^20^. Attributing the registered value to oneself further necessitates self-referential processes, namely forming a subjective belief that the feedback is a plausible depiction of one’s performance. Self-referential processes may engage components of the default-mode network (DMN), a brain network supporting internal mentation and social cognitive processes^21,22^; and particularly the ventromedial prefrontal cortex (VMPFC), which is also strongly associated with reward and valuation processes^23–25^. Indeed, previous neuroimaging studies found that both the VS and the VMPFC track the reward value of social feedback on former social performances and interactions^13,26^. With regards to updating of self-beliefs, VMPFC and VS guide optimistic updating of self-beliefs regarding the likelihood of encountering adverse future events^27^. Furthermore, VMPFC response to social approval correlates with subsequent updates in self-esteem^12,28^, which is strongly related to self-efficacy^29^. Moreover, psychopathologies that are marked by negative self-beliefs regarding competency such as depression and social anxiety are associated with dampened VS response to rewarding social feedbacks^30,31^. VS and VMPFC co-involvement was also previously associated with high trait self-esteem, during tasks that involve ascription of positive traits to oneself^32^ and viewing one’s own photo^33^.

The aforementioned neuroimaging studies have examined different forms of self-beliefs, but few studies investigated self-efficacy per se. Of those, the majority have so far mainly focused on neural correlates of self-efficacy as a trait (see ref. ^34^ for a review). One study that did examine how people update their self-efficacy found that in a simple response time task, VMPFC integrated information about previous success when people estimated future success^35^. Altogether, these findings link the processing of both non-social and social feedback positivity in the VS and VMPFC to a positive bias in updating self-related information and to psychopathologies that are marked by low self-efficacy. Yet, it remains to be determined whether individual differences in the function of the VS-VMPFC circuit underlie a diminished positive bias in how social feedback on actual performance shape self-beliefs regarding one’s performance—and particularly self-efficacy. Delineating the underlying mechanism of individual differences in self-efficacy updating could shed light on the neurobiological basis of a potential transdiagnostic feature of several psychopathologies^36^. This could ultimately inform process-based therapeutic interventions for mood and anxiety disorders.

**Fig. 1:**
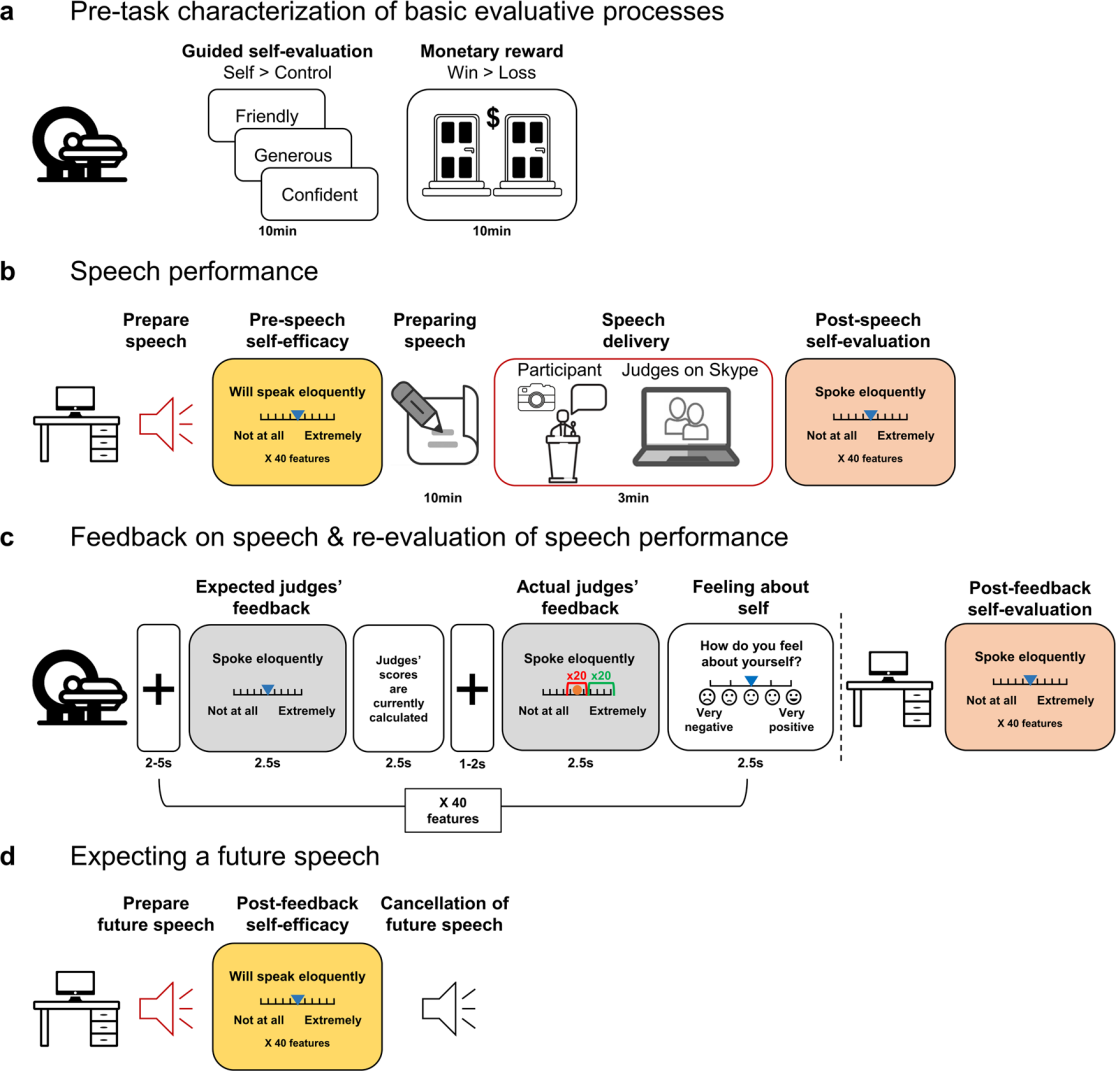
Experimental design. In this figure, a computer desk icon indicates the behavioral parts of the experiment, and the MRI icon indicates the fMRI session. **a** The first fMRI session of the experiment, during which participants completed a baseline assessment of brain activation in response to guided self-evaluation (attributing traits to the self vs. performing a lexical control task on the same words) and monetary reward (winning vs. losing money). **b** The first behavioral session of the socio-evaluative task, which included 3 min of public speaking in front of two judges in a pre-recorded video call and ratings of pre-speech self-efficacy (light orange) and post-speech self-evaluation (peach). Participants had 10 min to prepare the speech, and reported on their distress level at several time points relative to the speech onset (see Fig. 2a for the exact schedule of distress reports). **c** Exemplar trial from the social feedback task. Ranges of neutral and positive feedback are marked in red and green, respectively. After scanning, participants re-evaluated their speech performance. **d** Ratings of expected performance in an additional speech (i.e., post-feedback self-efficacy), which was ultimately canceled. All icons in this figure were downloaded freely from canva.com.

To address this gap, we developed a novel socially interactive experiment (Fig. 1) wherein healthy participants (*N* = 55) delivered a public speech—a common task for inducing social stress^37^—via a putative online video platform in front of two judges. During an fMRI scan that followed the speech, participants received fictitious and largely positive feedback on various aspects of their performance. Participants assessed their future performance (termed “self-efficacy” hereinafter) before giving the first speech and prior to an expected second speech that was ultimately not performed (this occurred outside the scanner after receiving feedback regarding performance on the first speech). In addition, participants evaluated their actual performance before and after the judges’ feedback on the delivered speech (termed “self-evaluation” hereinafter^4^). We used statistical modeling to capture individual differences in positive bias—that is, the degree to which participants relied on positive vs. neutral feedback when updating performance estimates of both self-efficacy and self-evaluation. We assume that self-evaluation and self-efficacy ratings are both forms of self-beliefs, in the sense that they constitute assessments that people make about their abilities and the outcomes they expect as a result of their efforts^38^. We expected that a smaller positive updating bias will be associated with trait social-affective sensitivities (i.e., symptoms and tendencies related to social anxiety, negative affect, depression, and low self-esteem^4,12^). We expected that higher neural response to positive feedback in VS and VMPFC and functional connectivity between these regions^32^, would be associated with greater positive updating bias regarding one’s performance.

## Methods

### Registered clinical trial

This interventional study was registered at clinicaltrials.gov under the NIH ClinicalTrials.gov identifier NCT03547713 on 06/06/2018.

### Participants

Fifty-eight healthy participants were initially recruited to this experiment (Mean_age_ ± SD: 25.17 ± 3.07 years, 35 females) through social media advertisement and following a screening procedure (*n* = 845 potential participants who met the inclusion criteria). The goal of the screening procedure was to assure the recruitment of participants with a wide range of social anxiety symptoms as indicated by the Liebowitz Social Anxiety Scale-Self-Report (LSAS-SR) questionnaire^39,40^, from about the same age range (21–35), and who also met the criteria for MRI scanning and spoke Hebrew at a mother-tongue level. Inclusion criteria also included at least 12 years of education, no reported history of psychiatric or neurological disorders (including ADHD) and no current use of psychoactive drugs. The screening form was completed in *qualtrics* and initiated by electronically signing an informed consent according to the Tel-Aviv University institutional review board (IRB). Next, potential participants completed self-report questionnaires addressing social anxiety (the LSAS-SR) and self-esteem^41^, provided demographic and medical details, and filled a standard MRI safety questionnaire.

On the experimental day, all participants who were eventually recruited to the experiment provided informed consent and received monetary compensation for their participation (50 NIS per hour). The study received ethical approval from the Tel-Aviv Sourasky Medical Center institutional review board (IRB), in accordance with the Declaration of Helsinki (committee reference number 0082-17-TLV). Three participants refused to perform the public speech, and 4 participants expressed strong disbelief in the experimental manipulation in the debriefing. One participant exhibited zero variability in his speech performance ratings. Thus, valid data for behavioral analysis were available for 50 participants (Mean_age_ ± SD: 25.24 ± 3.23, 33 females). In addition, two participants were removed from the fMRI analysis due to excessive head movements (see Functional Preprocessing below), thus leaving 48 participants (Mean_age_ ± SD: 25.29 ± 3.23, 31 females) eligible for analysis of fMRI data from the social feedback task. Note that although we did not conduct an explicit power analysis during study design, the final sample size was similar to that used in similar fMRI studies that examined brain-behavior correlations^12,28^.

### Procedure overview

At 1–2 days before the experiment, participants completed an online battery of questionnaires addressing social and affective sensitivities. On the experimental day, participants signed an informed consent and underwent a first MRI session (Fig. 1a) during which they completed anatomical scans and tasks probing neural processing of monetary reward and self-evaluation. Participants practiced these tasks before the scan for 15–20 min. Next, participants prepared a speech and conveyed it through a putative online video call outside the scanner (Fig. 1b). Before speech preparation and immediately after the speech, participants evaluated their expected and actual performance (termed “pre-speech self-efficacy” and “post-speech self-evaluation”, respectively) on 40 performance attributes (e.g., “eloquent”, “professional”, “interesting”), and continuously reported on their distress level. These speech performance evaluations were completed twice - once from a 1st-person point-of-view (POV; participants evaluation of their own performance) and once from the judges’ POV (participants evaluations of how the judges will rate their performance). We used the self-POV ratings to measure self-efficacy and self-evaluation, and the judges’ POV to measure the expected feedback following the actual performance. In another fMRI scan participants completed a social feedback task (Fig. 1c), during which they were reminded of their own evaluation of how they expected the judges would rate a specific attribute of their speech performance (i.e., the post-speech self-evaluation), and received corresponding feedback score from one of the judges for each of the 40 attributes. Judges’ scores were determined such that 50% of the feedbacks were neutral midpoints (between 4 and 6 on the 0–10 scale) and 50% were at the positive end of the scale (between 7 and 10), and participants did not know which judge gave them feedback on each trial. Participants also reported on their emotional experiences during and after the feedback. Afterward, participants re-evaluated their performance in the speech (“post-feedback self-evaluation”), recalled the judges’ feedback, and rated their expected performance in an upcoming second speech that was ultimately canceled (“post-feedback self-efficacy”; Fig. 1d). Finally, we conducted a semi-structured interview with participants whose main purpose was to indirectly probe suspicion about the manipulation.

### Public speaking task

The speech task was based on the Trier Social Stress Test paradigm^37^. We allotted participants 10 min to prepare a 5 min speech on their dream job and why they were well suited for it^4^. Preparation time was intentionally long, to increase the chances of giving a good performance that will match with the overall positive valence of the feedback. We told the participants that they would deliver the speech to a couple of experienced psychologists during a live video call via Skype, and devised a cover story that justified the conveyance of the speech in this online format. This cover story stated that the experimental question focused on how the physical vs. online presence of an audience affects public performance, and we told participants that they were assigned to the “online” experimental group. Participants were informed that the judges will evaluate their speech afterward, and that they will convey an additional speech later. We emphasized the importance of giving an engaging presentation, yet asked participants to refrain from referring questions to the audience. To further encourage participants to perform well, we also told them that the three best speeches will grant the speakers monetary prizes (350, 250, and 150 New Israeli Shekel [NIS]).

After speech preparation, the experimenter initiated the pre-recorded video call. Then, two confederates, one male in his late 30s and one female in her early 50s, introduced themselves as trained clinical psychologists with an expertise in interpersonal relations who work for a career counseling firm. Afterward, the judges instructed participants to begin the speech and kept an ambiguous facial expression for 3 min (instead of 5, to increase the chance that participants will talk the whole time), and then thanked the participant and terminated the call.

### Speech performance evaluation

Participants evaluated their expected and actual speech performance (termed “pre-speech self-efficacy” and “post-speech self-evaluation”, respectively) by referring to 40 performance attributes. The speech performance criteria were mostly derived from previous studies on public speaking^4,42–44^ and addressed various aspects of the performance (Supplementary Table 1). Twenty-nine items were positively valenced, and the remaining 11 negatively valenced items were reverse-coded in the analysis. Participants rated the quality of their performance on an 11-pt visual analog scale (VAS) ranging from “not at all” to “extremely”, without numerical indications, at four time points: (1) before the speech (“pre-speech self-efficacy”), (2) immediately after the speech (“post-speech self-evaluation”), (3) ~20 min after social feedback, where they re-evaluated the previous speech (“post-feedback self-evaluation”) and recalled the feedback score for each item, and (4) before the second speech (“post-feedback self-efficacy”; this immediately followed the previous stage). Note that in stages (1), (2), and (4), the speech questionnaire was administered twice, each time from either a self point-of-view (POV; e.g., “I appeared confident”) or the judges’ POV (e.g., “S\he appeared confident”). The two POVs were presented for two main reasons. First, during the social feedback task, we presented participants’ predictions about the judges’ evaluations, so it was more sensible to refer to the judges’ POV. Second, while previous studies addressed the self POV^4,13^, an open question that is not of interest here is whether social learning differs between the two POVs. Thus, in all behavioral analyses, we eventually focused on the self POV, except for the post-speech rating for which we used the judges’ POV. POV order was counter-balanced between participants. Stimuli presentation was randomized within each speech evaluation, and response time was always unlimited. Speech evaluations and the social feedback task were presented in PsychoPy (version 1.85). All texts and fixations in these tasks were presented in black color in the middle of the screen, overlaid on a gray background.

### Generation of social feedback valence in the social feedback task

Since we aimed to focus on positive valence and prevent spillover effects of negative valence, we incorporated only neutral and positive feedback, as done by others^16,30,44^. We thus set feedback scores such that they ranged from neutral (4–6 on the 11 pt VAS) to positive (7–10). We established the association of these ranges with neutral and positive valence in an independent experiment (Supplementary Results, Supplementary Fig. 1). The composition of feedback scores was as follows (score × amount): 4 × 6, 5 × 7, 6 × 7, 7 × 5, 8 × 7, 9 × 6, 10 × 2. The maximal score of 10 was presented only twice, in order to enhance the believability of the feedback. An algorithm coded in Python assigned neutral feedback to 20 items and positive feedback to the remaining 20 items in a partially randomized manner, as feedback assignment was constrained by a prediction error criteria. Specifically, the code attempted to maintain a believable and positively biased difference between the predicted and actual feedback scores (range [−2]–[+4]^13^). When it was not possible to meet this restriction due to an extreme score, the algorithm provided the next smallest possible error (e.g., [−3] or [+5]). The number of null prediction errors (i.e., when the feedback was equal to the expected score) was limited up to four occurrences for each participant^13^.

### Design of the fMRI social feedback task

Each trial started with a jittered fixation that lasted between 2 and 5 s (mean = 3.5 s). Then, one of the 40 performance criteria was presented for 2.5 s, while a reminder of the corresponding expected judges’ rating (i.e., “post-speech self-evaluation”) was marked on the VAS in a blue triangle. During the following anticipatory phase (2.5 s) the sentence “judges’ scores are currently calculated…” appeared on the screen. This was followed by another jittered fixation (1–2 s, mean = 1.5 s) and then the judges’ score appeared (2.5 s), marked by an orange circle. Lastly, participants rated how the current feedback made them feel about themselves on a 1–5 smiley-faces VAS through an MRI-compatible response box and by using their middle, index, and pinky fingers. Trials for the 40 items were presented randomly, apart from a restriction on no more than two consecutive presentations of feedback from the same condition. The onset of the first trial was preceded by a 22.5 s long fixation (10 TRs). The total task duration was 10:15 min, and it was performed in a single run. Prior to the MRI session, we instructed participants about the task and let them practice it until they got well accustomed with its pace. The practice was performed on three items that were not included in the 40 speech evaluation criteria.

### Assessment of state emotions

**Fig. 2:**
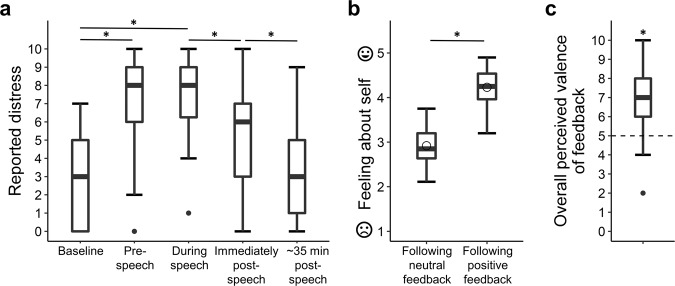
Validation of stress induction and emotional impact of positive feedback. **a** Subjective distress ratings collected at 5 time-points relative to speech performance, ranging from a baseline measurement prior to task announcement and up to ~35 min post-speech. Note that the third and fourth ratings were both collected immediately after the speech. **b** Participants’ ratings of their feelings about themselves (i.e., their response to the question “how does the feedback make you feel about yourself?” that appeared on each trial) following positive vs. neutral feedback. **c** Overall perceived valence of judges’ feedback. The statistical test compared the value of the median valence rating to the midpoint of the scale, which is marked by a dashed line. In all boxplots, the horizontal line marks the median, and the upper and lower bounds of the box denote the 75th (Q3) and 25th (Q1) percentiles, respectively. The line stretches between the minimum (Q1−1.5*interquartile range) and maximum (Q3 + 1.5*interquartile range) of the data. The empty circle, when presented for variables on which we performed parametric tests, indicates the mean. Asterisks denote significance: *p < 0.001.

For the purpose of basic task validation, we sought to verify that the public speaking task induced a subjective experience of distress; and that the social feedback in general was experienced positively. Induction of distress by the speech was assessed through subjective ratings that participants provided on an 11-pt VAS ranging from “not at all” to “extremely”, without numerical indications, at 5 time points (Fig. 2a). In addition, after the feedback task participants rated their overall experience of the feedback (from “very negative” to “very positive”) using a similar VAS.

### Auxiliary fMRI tasks for characterizing basic evaluative processes

To test whether the processing of judges’ feedback positivity involved activating neural correlates of reward and self-evaluation, participants completed two auxiliary fMRI tasks that were designed to evoke neural representations of these processes. Self-evaluation was assessed with a modified version of the self-referential encoding task (SRET)^45^. In this block-design task, participants judged if 64 social traits were descriptive of them or not on “self” blocks, or performed a lexical decision task on the same traits during “control” blocks (deciding whether the first two letters of the trait were presented in the alphabetical order). Each block was initiated with an instruction screen (2.5 s, 1 TR) which informed participants about the task they had to perform in the forthcoming block (i.e., self vs. control). Afterward, a set of 4 traits was presented for 12.5 s (5 TR), such that each trait appeared for 3.125 s. During this phase, participants used an MRI-compatible response box to provide yes/no answers while each trait appeared, by using their middle and index fingers, respectively. This was followed by a fixation that lasted 2.5 s. The task consisted of 32 blocks in total, which were performed in two separate runs. Each run contained 16 blocks and started with a 22.5 s long fixation (10 TRs), and lasted 4:55 min in total. Note that the traits in the SRET were sorted into four conditions based on a categorization of their valence and social domain. However, this distinction was not relevant for the current study, and thus we averaged the self > control contrast across conditions. The experiment was programmed and presented in PsychoPy (version 1.85).

Reward responsiveness in the VS was probed using a guessing task which is known to elicit robust activation in the VS^46^. In this event-related paradigm, participants selected one out of two doors that led to either monetary prizes or losses. Specifically, each trial started with a white fixation cue presented in the center of a black screen (500 ms). Then, two identical doors were presented side-by-side for 4000 ms. Participants were instructed before the task that behind one of the doors was a monetary prize [+5 NIS, currently equivalent to ~$1.4] whereas behind the other door there was a loss (−2.5 NIS). Participants were told that if they did not make their choice while the doors were presented, the computer would select a door randomly. Participants used an MRI-compatible response box to choose one of the doors. Then, after another fixation cue (500 ms), a feedback screen was displayed (1000 ms) wherein a green arrow indicated a correct guess leading to a monetary prize, and a red arrow indicated monetary loss. Finally, a blank black screen jittered inter-trial interval occurred between each trial (1500–14,000 ms, *M* = 4000 ms). The task consisted of 60 trials with 30 predetermined wins and 30 losses presented in a pseudorandom order and divided equally into two functional runs. That is, unknown to participants, their choice did not influence whether a trial was a win or loss. Prior to the task, participants were instructed about its stages and were informed that the size of the monetary prize will eventually be determined by summing the monetary gains and losses from 12 (out of 60) randomly chosen trials, so that they could earn up to 60 NIS. The experiment was programmed and presented using E-prime software (Psychology Software Tools, Pittsburg, PA). The total task duration was 585 s.

### Analysis of subjective ratings of distress, emotions, and speech performance

We used non-parametric tests for examining speech-induced distress (Friedman’s ANOVA) and the overall assessment of feedback valence that followed the task (Wilcoxon test), since these were both ordinal and non-normally distributed variables. To assess the momentary effect of positive vs. neutral feedback on self-related feelings, we averaged the subjective ratings during these two conditions and submitted the mean values to a paired-samples *t*-test. To explore the changes in ratings of self-efficacy and self-evaluation regarding speech performance throughout the experiment, we submitted the mean ratings to a one-way repeated measures ANOVA and conducted post hoc pairwise comparisons using paired-samples *t*-tests. All statistical analyses were carried with IBM SPSS statistics 20. All reported *p*-values in the study are two-tailed. The effect size of Friedman’s ANOVA was estimated using Kendall’s W. Effect size of all Wilcoxon tests was r, which is calculated as the z statistic divided by the square root of the sample size. Effect sizes of all t-tests were estimated using Cohen’s d via the *effsize* package implemented in r (https://github.com/mtorchiano/effsize/).

### Statistical modeling of speech performance ratings

In order to examine the influence of judges’ feedback on self-evaluation and self-efficacy ratings, we modeled the relationship between feedback and the different types of ratings within a hierarchical Bayesian framework as the covariance of a multivariate normal probability distribution. Thus, we could parametrically estimate how a participant’s self-ratings were influenced by feedback while controlling for the participant’s other self-ratings. Note that the multivariate normal probability function is not the most appropriate model for our data, since we used discrete ratings that come from a limited scale, whereas a normal distribution is continuous and unlimited. A proper model would treat any value above/below some threshold as the maximum/minimum rating. Yet, such a model would require either the Beta Cumulative Distribution Function (CDF)^47^, whose implementation demanded an unfeasible computational duration; or the multivariate normal CDF function that requires complicated computations and is not available in statistical estimation platforms known to us. Thus, we made a simplifying assumption that the ratings are directly drawn from normal distributions.

We tested two alternative models, which both described the series of 5 speech scores—namely pre-speech self-efficacy (*y*_1_), post-speech self-evaluation (*y*_2_), the judges’ feedback (*y*_3_), post-feedback self-evaluation (*y*_4_), and post-feedback self-efficacy (*y*_5_)—as drawn from a multivariate normal distribution. We included each rating type in the model, since this was necessary for cleanly assessing the impact of feedback on post-feedback scores, i.e., by removing variance in these scores that were explained by other ratings. For instance, post-speech self-evaluation could impact post-speech self-efficacy regardless of what was the feedback, and therefore we included it in the model. The first model ascribed differential influence to positive vs. neutral feedback scores (i.e., 7–10 vs. 4–6 on the 0–10 scale, respectively) by estimating different covariance matrices. Thus, the relation between scores (*y*_1:5_) for a specific question (indexed by *q*) was determined by a subject-level (indexed by *s*) covariance matrix Σ_*s*_, which was different for questions that received positive (abbreviated “pos”) and neutral (abbreviated “neu”) feedback:

1 $$\begin{array}{l}\left[ {y_{1:5_{s,q}}} \right]\sim {{{\mathcal{N}}}}\left( {\left[ {\mu _{1:5_s}} \right],{{\Sigma }}_{Pos_s}} \right)\\ for\;y_{3_{s,q}} \ge 7\\ \left[ {y_{1:5_{s,q}}} \right]\sim {{{\mathcal{N}}}}\left( {\left[ {\mu _{1:5_s}} \right],{{\Sigma }}_{Neu_s}} \right)\\ for\;y_{3_{s,q}} \,<\, 7\end{array}$$

Note that to simplify the behavioral model, we only allowed it to have two covariance matrices (as opposed to a spectrum of covariance matrices, which would be required for a parametric approach). In the equation above, each score is drawn from a subject-level distribution with a mean value of *μ*_*s*_—except for the mean judges’ score which was known and fixed for all participants. Each Σ_*s*_ (i.e., for either positive or neutral conditions) is a subject-level covariance matrix whose diagonal describes the variance of each score, and the off-diagonal elements describe the coupling between each pair of scores. Each Σ_*s*_ was constructed as a product of a correlation matrix Ω_*s*_ and a vector of coefficient scales *τ*_*s*_ as follows:

2 $${{\Sigma }}_s = \left[ {\begin{array}{*{20}{c}} {\tau _{s1}} & 0 & 0 \\ 0 & \ddots & \vdots \\ 0 & \ldots & {\tau _{s5}} \end{array}} \right] \times {{\Omega }}_s \times \left[ {\begin{array}{*{20}{c}} {\tau _{s1}} & 0 & 0 \\ 0 & \ddots & \vdots \\ 0 & \ldots & {\tau _{s5}} \end{array}} \right]$$

Thus, our main parameters of interest resided in ${{\Omega }}_{\rm{Pos}_s}$ and ${{\Omega }}_{\rm{neu}_s}$, which are 5 × 5 correlation matrices where each cell contains a parameter representing the correlation between a specific pair of score types. To assess the influence of the feedback, we examined specifically those correlation parameters that represent the coupling between judges’ feedback and the two sorts of post-feedback ratings—the post-feedback self-evaluation and self-efficacy ratings. Specifically, we focused on the cells indicating the correlation between the judges’ scores and post-social feedback ratings on positive vs. neutral feedback trials. We quantified positive update bias as the difference between the feedback-rating correlations given positive and neutral feedback as follows:

3 $${\rm{self}\;evaluation\;\rm{update}\;\rm{bias}} = {{\Omega }}_{\rm{Pos}_s(y_3,y_4)} - {{\Omega }}_{\rm{Neu}_s(y_{3,}y_4)}$$

4 $${\rm{self}\;\rm{efficacy}\;\rm{update}\;\rm{bias}} = {{\Omega }}_{\rm{Pos}_s(y_3,y_5)} - {{\Omega }}_{\rm{Neu}_s(y_{3,}y_5)}$$

The second model assumed that positive vs. negative deviance (i.e., prediction error; PE) between the expected feedback (i.e., post-speech self-evaluation) and the actual judges’ scores, had a differential influence on post-feedback ratings^4^. As in the first model, this model estimated distinct covariance matrices for sores within positive vs. neutral feedback, yet ‘positive’ and ‘neutral’ were defined based on PE rather than on absolute score. For questions with PE = 0, priors of Σ_*s*_ were defined based on the average of the parameters estimated for the positive and negative PE questions.

An elaborated description of the hierarchical structure of the models, including all of the hyperpriors that were placed on subject- and group-level parameters, are presented in the Supplementary Code.

### Model fitting and model comparison

We used the RStan package (Stan Development Team, 2016. RStan: the R interface to Stan. R package version 2.19.2), which utilizes Markov chain Monte Carlo (MCMC) sampling algorithms, to fit the models. We fit each model with four independent MCMC chains using 10,000 iterations after 1500 iterations for initial algorithm warmup per chain and a thin factor of 4, thus resulting in 10,000 valid posterior samples. Maximum tree depth was 10, and adapt delta was set at 0.9 and raised up to 0.99 to eliminate divergent transitions. We assessed the convergence of MCMC chains to the target distributions by inspecting R-hat values (should be smaller than 1.1) and effective sample sizes (should be >1000) for all model parameters^48^, by using the ShinyStan package (version 2.5.0). We compared candidate models by implementing K-fold cross-validation with 10 folds. Each model fit (i.e., within each fold) was sampled with parameters identical to those mentioned above, except that 5000 iterations were performed instead of 10,000—thus resulting in 5000 valid posterior samples per fold. Predictive accuracy of each model was calculated according to the expected log pointwise predictive density (ELPD) by using the LOO package in R^49^. ELPD indicates the height (density) of the probability distribution at data points that were held-out, given the model parameters. A difference in ELPD that was at least twice the size of the standard error of the estimated difference, indicated a reasonable evidence for preference of the better-performing model^50^.

The performance of the winning model was also assessed using a posterior predictive check, wherein we examined the correlation between the update bias indices (equations (3)–4)) and the corresponding actual difference between Pearson correlation coefficients describing the relation between the judges’ scores and the relevant post-feedback score under positive vs. neutral conditions. In additional posterior predictive checks, we assessed if the correlations between pre- and post-feedback scores in the actual data, corresponded with those in a simulated dataset that was generated based on parameters estimated by the model (Supplementary Results).

### Assessment of positive bias at the group level

In order to assess positive bias at the group level, we computed the median of the relevant Ω parameters across participants on each iteration of the model, thus resulting in a distribution of 10,000 posterior samples of group-level medians for each parameter. We then checked if the 95% highest-density intervals (HDI) of the distributions resided beyond 0 and on the positive side of the scale.

### Controlling for individual differences in prediction error

The experimental design was unbalanced in terms of positive and negative prediction errors. Thus, to ensure that positive update bias indices did not reflect incidental individual differences in the degree to which positive, as compared to neutral, feedback deviated from participants’ expectations (i.e., “prediction error”), we regressed out the influence of this deviation difference on all quantified biases. This deviation difference was computed by subtracting between the mean of prediction errors during positive vs. neutral trials.

### Integrative profiling of symptoms and correlation with model parameters

Participants completed a battery of questionnaires that could potentially relate to diminished positive bias. Questionnaires of social anxiety included the LSAS-SR^39^ and the Social Phobia Inventory (SPIN)^51^. Questionnaires addressing biased processing of both negative and positive social feedback included the Brief Fear of Negative Evaluation Scale (BFNE)^52^, the Fear of Positive Evaluation Scale (FPES)^53^ and the Disqualification of Positive Social Outcomes Scale (DPSOS)^54^. The DPSOS has two subscales—a “self” subscale that assesses one’s tendency to attribute personal successes to himself; and a “other” subscale, which assesses one’s tendency to attribute positive social experiences such as social praise to external social factors rather than to his/her own effort or ability (e.g., laughing from one’s joke because it’s the polite thing to do). Self-esteem was assessed with Rosenberg’s Self-esteem scale (RSE; note that this questionnaire was administered only during screening)^41^. Negative affect was measured using the State-Trait Anxiety Inventory (STAI)^55^ and the neuroticism subscale from the Neuroticism-Extroversion-Openness—Five Factor Inventory (NEO-FFI)^56^. Depression and motivational tendencies were measured using the Beck Depression Inventory (BDI-II)^57^ and the Sensitivity to Reward and Punishment Questionnaire (SPSRQ)^58^, respectively. Note that the SPSRQ has two subscales, which differently assess reward and punishment sensitivity. To obtain an integrative and dimensional profiling of individual differences, we performed a principal component analysis (PCA) on the questionnaires’ scores using SPSS20. We used varimax rotation and extraction of factors was based on Eigenvalues >1. Individual differences in scores on the emerging components were correlated with the positive bias parameters using Pearson correlations. Before examining these correlations, we regressed out the influence of between conditions' mean prediction error difference on these components.

### Functional magnetic resonance imaging (fMRI) data acquisition

All scans were performed using a Siemens 3 T Prisma Magnetom VD13 echo speed scanner with a 20-channel head coil located at the Wohl Institute for Advanced Imaging at the Tel-Aviv Sourasky Medical Center. Structural scans included a T1-weighted 3D axial spoiled gradient echo (SPGR) pulse sequence (repetition time/echo time [TR/TE] = 1860/2.74 ms, flip angle = 8°, voxel size = 1 × 1 × 1 mm, field of view = 256 × 256 mm, slice thickness = 1 mm).

Functional whole-brain scans of all fMRI tasks were performed in an interleaved bottom-to-top order with a T2*-weighted gradient echo planar imaging pulse sequence (TR/TE = 2500/30 ms, flip angle = 82°, voxel size = 2.3 × 2.3 × 3 mm, the field of view = 220 × 220 mm, slice thickness = 3 mm, 42 slices per volume). For three participants in the social feedback task and the guided self-evaluation task, the TE was 35, flip angle was 90° and 38 slices per volume were obtained. These participants were included in the analysis. The number of volumes acquired for each of the fMRI tasks was as follows: social feedback—246; guided self-evaluation—2 sessions including 118 volumes each; reward—2 sessions including 117 volumes each.

### Anatomical preprocessing

Raw DICOM data images were converted to NIFTI format and organized to conform to the ‘Brain Imaging Data Structure’ specifications (BIDS)^59^. Preprocessing was conducted using FMRIPREP version 1.5.8^60^, a Nipype based tool^61^. Within the FMRIPREP framework, each T1-weighted (T1w) image was corrected for intensity non-uniformity (INU) using ‘N4BiasFieldCorrection’ v2.1.0, distributed with ‘AntsApplyTransforms‘ (ANTs version 2.2.0). The T1w reference was then skull-stripped with a Nipype implementation of the ‘antsBrainExtraction.sh‘ workflow (from ANTs), using OASIS30-ANTs as a target template. Brain tissue segmentation of cerebrospinal fluid (CSF), white matter (WM), and gray matter (GM) were performed on the brain-extracted T1w using ‘FAST‘ (FSL version 5.0.9). A T1w-reference map was computed after registration of the INU-corrected T1w image using ‘mri_robust_template‘)FreeSurfer version 6.0.1). Volume-based spatial normalization to one standard space (MNI152NLin2009cAsym) was performed through nonlinear registration with ‘antsRegistration‘ tool of ANTs version 2.2.0, using brain-extracted versions of both T1w reference and the T1w template. The ICBM 152 nonlinear Asymmetrical template version 2009 was selected for spatial normalization.

### Functional preprocessing

First, a reference volume and its skull-stripped version were generated using a custom methodology of FMRIPREP, and the susceptibility distortion correction (SDC) was omitted. The BOLD reference was then co-registered to the T1w reference using ‘MCFLIRT‘ (FSL version 5.0.9) with the boundary-based registration cost function. Co-registration was configured with nine degrees of freedom to account for distortions remaining in the BOLD reference. Head-motion parameters with respect to the BOLD reference (transformation matrices, and six corresponding rotation and translation parameters) were estimated before any spatio-temporal filtering. BOLD runs were slice-time corrected using ‘3dTshift‘ from AFNI version 16.2.07, and their time-series were resampled onto their original, native space by applying the transforms to correct for head-motion. Several confounding time-series were calculated based on framewise displacement (FD), DVARS and three region-wise global signals (extracted within the CSF, the WM, and the whole-brain masks). In addition, a set of physiological regressors were extracted to allow for component-based noise correction (CompCor). Principal components were estimated after high-pass filtering of the pre-processed BOLD time-series (using a discrete cosine filter with 128 s cut-off) for the two CompCor variants: temporal (tCompCor) and anatomical (aCompCor). Six tCompCor components were then calculated including only the top 5% variable voxels within that subcortical mask. For aCompCor, six components were calculated within the intersection of the subcortical mask, and the union of CSF and WM masks calculated in T1w space, after their projection to the native space of each functional run. For each CompCor decomposition, the k components with the largest singular values were retained, sufficient to explain 50% of variance across the nuisance mask. The remaining components were dropped from consideration. All re-samplings were performed with a single interpolation step by composing all the pertinent transformations. Gridded (volumetric) re-samplings were performed using ANTs, configured with Lanczos interpolation to minimize the smoothing effects of other kernels, while non-gridded (surface) re-samplings were performed using ‘mri_vol2surf‘ (FreeSurfer). Many internal operations of FMRIPREP use ‘Nilearn‘, principally within the BOLD-processing workflow (for more details of the pipeline, see https://fmriprep.readthedocs.io/en/stable/workflows.html).

Eventually, the confounds file in all 1st-level fMRI analyses included the following regressors: the time series derived from head motion estimates, their quadratic terms, and the temporal derivatives of both series (a total of 24 regressors); the standard deviation of DVARS; the 6 aCompCor components; and framewise displacement (FD). Note that frames that exceeded a threshold of 0.9 mm FD were annotated as motion outliers^62^. Finally, spatial smoothing of the data was performed using SPM12 (full-width at half-maximum: 6 mm). Data of two participants were excluded from the fMRI analysis of the social feedback task due to head movements that exceeded 3 mm.

### Statistical analysis of fMRI data from the social feedback task

Statistical analysis of the fMRI data was conducted with Statistical Parametric Mapping software (SPM12; http://www.fil.ion.ucl.ac.uk/spm). We implemented a general linear model (GLM) in order to estimate neural responses to the experimental conditions.

In the fMRI analysis, we first aimed to establish that positive feedback from judges activated our key regions of interest; the VS and VMPFC (known to process reward- and self-evaluation, respectively). For this purpose, we modeled the response to social feedback with a parametric regressor that captured the encoding of the positivity of judges’ feedback by detecting brain regions in which brain activity correlated linearly with feedback scores in the neutral to positive range^13^. Respectively, in the 1st-level GLM we created four predictor variables for the different task periods as follows: (1) reminder of expected feedback, (2) anticipation, (3) reception of social feedback, (4) rating of feeling about the self. In addition, we incorporated the following three parametric modulators: (5) expected feedback score, which was aligned with (1), (6) actual feedback score, which was aligned with (3), and (7) the number of button presses during the rating phase, which was aligned with (4) and modeled in order to account for motor activation. All parametric modulators were mean-centered and orthogonalized. The duration of all conditions was 2.5 s. All predictors were convolved with a canonical hemodynamic response function, and data were subjected to SPM12 default high-pass filter cutoff (128 s). We added confound regressors to participants’ 1st-level model as well (see “Functional Preprocessing” above).

Coefficients estimated for each participant for the parametric regressor of the actual feedback score during feedback reception were submitted to a 2nd-level one-sample *t*-test in SPM12. The resulting activation map was thresholded with a voxel-level threshold of *p* < 0.001 and a cluster-level corrected qFDR < 0.05. The primary voxel-level threshold of *p* < 0.001 has previously been suggested to control well for the false-positive rate^63^. This threshold was set for all upcoming whole-brain analyses of fMRI data as well.

### Definition of regions of interest (ROI)

We defined ROIs in the bilateral VS and VMPFC, which we used in all upcoming analyses of fMRI data from the social feedback task, as follows. For the VMPFC we used regions 41–42 from the Brainnetome atlas^64^. We chose these regions due to their high probability of association with reward tasks in the atlas, and their overlap with VMPFC locations from prominent meta-analyses on positive subjective value^25^ and self-referential processing^22^. Moreover, the group-level peak activation for the judges’ feedback positivity contrast (*x* = 7, *y* = 42, *z* = −16) was located within these VMPFC clusters. For the VS we used nucleus accumbens clusters from a novel atlas of striatum components^65^, which we found to cover the VS more accurately than the VS ROIs in the Brainnetome atlas.

### Covariance of self-efficacy update bias with brain encoding of social feedback positivity

To test the correlation of self-efficacy update bias with brain activity during encoding of social feedback positivity, we conducted a 2nd-level multiple regression in SPM12. In this analysis, we entered between-subject covariates for the individual differences in self-efficacy update indices, as well as for between conditions differences in mean prediction errors, which are known to affect brain activity in our key ROIs—the VS and VMPFC^12,66^. We restricted this analysis to our main ROIs by combining the above-defined VS and VMPFC into one mask. We then searched for positive and negative correlations of self-efficacy update bias with activation of voxels within the masked brain area, by setting a statistical threshold of voxel-level *p* < 0.001 SVC FWE < 0.05. Additional exploration of correlations with self-efficacy update bias were conducted at the whole-brain level (i.e., without using a mask).

### Statistical analysis of fMRI data from the auxiliary fMRI tasks

Analyses of these tasks were performed by implementing GLMs in SPM12 as well. In the 1st-level model of the monetary reward task, we created predictor variables for the four phases of the task as follows: (1) choice (2.5 s), (2) anticipation (2.5 s), win (i.e., reward; 1 s) and loss (i.e., punishment; 1 s). The “win” and “loss” phases were aligned with the onset of the outcome phase. The period prior to outcome onset was divided into two epochs: The first TR (lasting 2500 ms) following the doors onset was classified as “choice”, and the following TR was coded as “anticipation”^67^. We then computed linear contrasts for win>loss, which represented sensitivity to reward versus punishment.

The 1st-level GLM of the guided self-evaluation task was estimated using the following eight predictors: (1–4) predictors for the four task conditions in their “self” condition; (5–8) predictors for the matching “control” conditions. The event duration of each block was 12.5 s. We then computed the main linear contrasts of interest, wherein we summed all “self” block and contrasted them against the sum of all “control” blocks.

For both tasks, confound regressors from the preprocessing step were included as covariates of no interest in the 1st-level GLM, and were identical to those used for the social feedback task (see “Functional Preprocessing” above).

### Representational similarity analysis (RSA)

To test whether the processing of judges’ feedback positivity involved activating neural correlates of reward and self-evaluation, we performed a searchlight representational similarity analysis (RSA) between the social feedback task and auxiliary fMRI tasks that probed each of these processes. RSA is suitable for revealing similarities in multivoxel encoding of affective and cognitive processes across tasks and mental states^45,68^.

We conducted a searchlight RSA between the social feedback task (parametric regressor of the social feedback’s valence>baseline contrast) and each of the auxiliary tasks assessing reward (win > loss contrast) and guided self-evaluation (self > control contrast). The 3 first participants did not complete the reward task, so valid fMRI data were available for 45 participants in this task (Mean_age_ ± SD: 25.27 ± 3.25 years, 29 females).

Searchlight RSA was performed using non-smoothed fMRI data via the RSA toolbox^69^, while defining Spearman’s rank correlation as the distance measure. The searchlight RSA was performed with a sphere with a radius of 9 mm (volume: ~3200 mm³), which covered a radius of ~3–4 voxels around the sphere’s center^70^. Throughout the process of searchlight RSA, the sphere is centered around each voxel in the brain one step at a time, and that voxel receives the value of similarity between the two contrasts within the sphere. After performing the searchlight, we fisher-z transformed the similarity values in each voxel and submitted them to a 2nd-level one-sample t-test via SPM12 in order to determine group-level significance^45^.

### Psychophysiological Interaction (PPI) analysis

To examine whether VS connectivity altered during feedback reception as a function of judges’ feedback positivity, we conducted a Psychophysiological Interaction (PPI) analysis. PPI analysis was conducted using CONN 18b^71^. During the setup phase in CONN we imported the fMRI data that was pre-processed in *fMRIPrep*, yet we performed additional pre-processing steps as follows. The confound regressors were similar to those used in the neural activation GLMs, apart from the aCompCor regressors which we re-computed in CONN. Masks of white matter, gray matter and cerebrospinal fluid were produced by performing a segmentation on the structural image of each subject. During the denoising step, the following confounds were regressed out: (1) the first five principal components of the CSF and white matter signals, based on the aCompCor method, and (2) task-related BOLD signals by performing linear de-trending. Bandpass temporal filtering was performed to remove slowly fluctuating signal (0.008 Hz) such as scanner drift.

In the 1st-level analysis, we defined the social feedback reception condition and its corresponding parametric modulation by feedback score as conditions of interest in the setup phase. We then computed a seed-to-voxel PPI analysis using the bilateral VS as a seed region. The PPI GLM included a regressor of the physiological variable (i.e., time course of activity in the seed ROI), and two regressors representing the interaction of the time series of the seed ROI with the experimental conditions. PPI values in each voxel were computed using bivariate regression and were converted to z-scores using Fisher’s z-transformation. We examined the whole-brain connectivity group-level effects by computing a 2nd-level analysis in CONN. To examine the association of VS-to-VMPFC connectivity with a positive bias, we performed a 2nd-level multiple regression analysis in CONN. As in the brain activity analysis, we entered covariates for individual differences in self-efficacy update indices and for between conditions differences in mean prediction error. Yet, here we restricted the regression analysis to a mask covering only the bilateral VMPFC. Subsequent additional explorations of correlations with self-efficacy update bias were conducted at the whole-brain level as well. In an exploratory analysis, we tested the role of VS functional connectivity in mediating VS activity-related changes in self-efficacy update bias (see Results). To this end, we ran a mediation analysis using a bootstrapping approach with 5000 samples using the PROCESS v3.5 macro in SPSS^72^.

## Results

### Affective impact of the public speech delivery and related social feedback

We first sought to validate the main components of our novel experimental setup. As expected, distress levels increased immediately before and during speech delivery compared to baseline levels (i.e., prior to speech announcement), and then returned to baseline level ~35 min following speech delivery (Fig. 2a; main effect of time-point on self-reported distress via Friedman’s ANOVA: *χ²*(4) = 219.20, *p* < 0.001, Kendall’s *W* = 0.65). Feelings about the self after positive feedbacks (Mean ± SD: 4.23 ± 0.37) were more positive than those reported after neutral feedbacks (Mean ± SD: 2.92 ± 0.40; Paired samples *t*-test: (49) = 19.69, *p* < 0.001, *d* = 3.40, 95% CI = [1.18, 1.45]); Fig. 2b). In addition, the overall valence of the entire feedback was perceived as positive (median = 7), relative to the mid-point of the scale (hypothetical median = 5; Wilcoxon one-sample signed-rank test: *Z* = 5.41, *p* < 0.001, *r* = 0.77; Fig. 2c).

### The impact of positive social feedback on self-evaluation and self-efficacy assessments

**Fig. 3:**
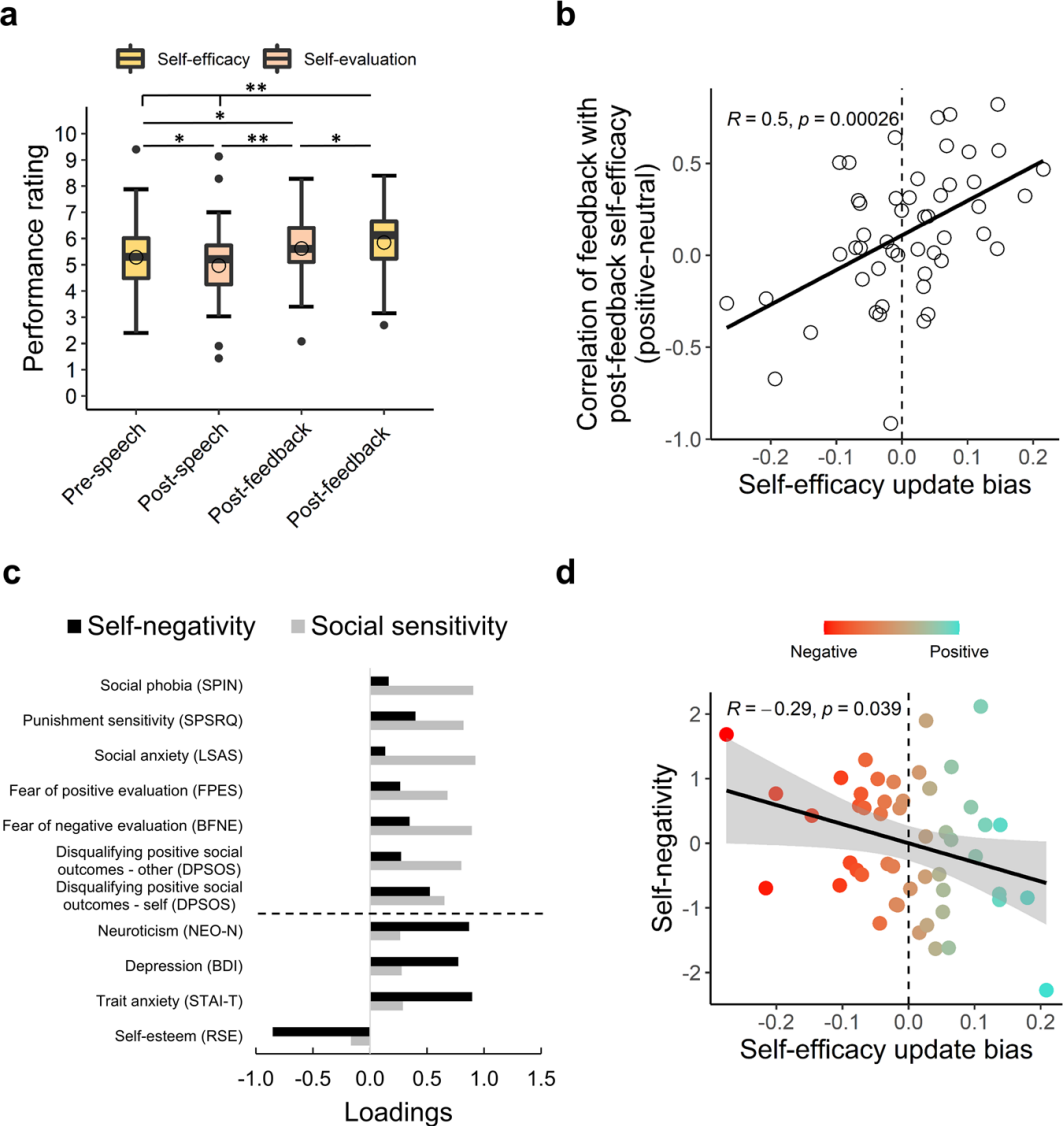
Self-evaluation and self-efficacy updating, and their association with psychopathological symptoms. **a** Ratings of self-evaluation (light orange) and self-efficacy (peach) collected throughout the experiment. In all boxplots, the horizontal line marks the median, and upper and lower bounds of the box denote the 75th (Q3) and 25th (Q1) percentiles, respectively. The line stretches between the minimum (Q1-1.5*interquartile range) and maximum (Q3 + 1.5*interquartile range) of the data. The empty circles indicate the mean. Asterisks denote significance: **p < 0.001; *p < 0.05. **b** Posterior predictive check of modeled self-efficacy update bias. The scatterplot depicts the correspondence between self-efficacy update bias and within-subject correlation of feedback and post-speech self-efficacy rating for positive minus neutral feedback. **c** Loadings of questionnaires on the “Self-negativity” (black) and “Social sensitivity” (gray) components. The black dashed line differentiates between questionnaires that loaded highly onto each component. See “Methods” for questionnaires’ abbreviations. **d** A scatterplot depicting the correlation between self-negativity and self-efficacy update bias. The dots are colored with a red (anti-positive) to turquoise (positive) color scaling denoting self-efficacy update bias.

We next aimed to determine how participants’ assessments on their self-efficacy (expected performance) and self-evaluation (actual performance) were affected by the social feedback given during the fMRI task. Overall, ratings were positively updated following the social feedback task (repeated-measures ANOVA on the two self-efficacy and two self-evaluation ratings collected throughout the task, the main effect of time: *F*(3) = 18.58, *p* < 0.001, *µ*² = 0.28; Fig. 3a). Post hoc pairwise comparisons revealed that both self-evaluation and self-efficacy increased following social feedback reception (post-feedback self-evaluation [M ± SD: 5.62 ± 1.04] vs. post-speech self-evaluation [M ± SD: 4.97 ± 1.38]: t(49) = 5.22, *p* = <0.001, *d* = 0.5, 95% CI = [0.39, 0.89]; post-feedback self-efficacy [M ± SD: 5.85 ± 1.3] vs. pre-speech self-efficacy [M ± SD: 5.28 ± 1.29]: t(49) = 4.55, *p* < 0.001, *d* = 0.43, 95% CI = [0.31, 0.81]; pFDR < 0.05). This suggests that the feedback had a positive influence on performance evaluations, and that participants expected to perform better in an upcoming second speech.

### Statistical modeling of self-assessment updates

We next examined the influence of judges’ feedback on self-evaluation and self-efficacy ratings, which we modeled within a hierarchical Bayesian framework. Model comparisons revealed that the model that ascribed differential influence to positive vs. neutral feedback on post-feedback ratings (i.e., in terms of the absolute valence of feedback; Eq. (1)), outperformed another plausible model that accounted for the differential influence of positive vs. negative prediction errors (Supplementary Table 2). However, note that the design was unbalanced in terms of positive and negative prediction errors. Both models showed adequate convergence of the Markov Chain Monte Carlo (MCMC) chains, and a posterior predictive check confirmed that the parameters of the winning model associated with linear correlations within participants’ actual scores (Fig. 3b and Supplementary Fig. 3).

We next examined the two positive update bias indices that we computed based on the parameters of the model, namely “self-evaluation update bias” (Eq. (3)) and “self-efficacy update bias” (Eq. (4)). Note that the two update bias indices were uncorrelated (*r*(48) = 0.06, *p* = 0.68), thereby suggesting that these are two distinct measures. After extracting positive bias parameters, we tested whether positive bias manifested at the group level. Contrary to previous studies^4,13^, we did not find evidence for a group-level positive bias in updating of either self-evaluation (median = 0.021; 95% HDI: [−0.03, 0.07]) or self-efficacy (median = 0.007; 95% HDI: [−0.05, 0.06]; see Supplementary Fig. 4 for posterior distributions of the parameters).

We subsequently tested the association of update bias with trait affective-social psychopathological tendencies that are linked to low self-efficacy and negatively biased processing of social feedback. We conducted a principal component analysis (PCA) on the battery of delivered questionnaires (see “Methods”, Fig. 3c and Supplementary Table 3), and then correlated the emerging components with participants’ update bias. The PCA revealed three components: one component, termed “Social sensitivity” (explained variance = 57.2%, eigenvalue = 6.86), primarily reflected symptoms of social anxiety and sensitivity to social evaluation. Another component, termed “Self-negativity” (explained variance = 14.64%, eigenvalue = 1.76), reflected symptoms of anxiety, depression and low self-esteem. A third component (explained variance = 9.02%, eigenvalue = 1.08) was affected only by one questionnaire and was excluded from further analysis.

We next tested the correlation between the social sensitivity and self-negativity components and the two types of update bias (i.e., a total of four possible correlations). We found that participants with higher self-negativity exhibited a less positive self-efficacy update bias (*r*(48) = −0.29, *p* = 0.039 uncorrected, 95% CI: [−0.53, −0.02]; Fig. 3d). Self-negativity was not related to self-evaluation update bias (*r*(48) = −0.19, *p* = 0.19, 95% CI: [−0.45, 0.09]). Social sensitivity did not correlate significantly with neither of the biases (self-evaluation: *r*(48) = 0.14, *p* = 0.35, 95% CI: [−0.15, 0.4]; self-efficacy: *r*(48) = −0.14, *p* = 0.35, 95% CI: [−0.4, 0.15]). Thus, participants who had higher scores on a psychopathological dimension combining symptoms of anxiety and depression alongside low self-esteem, updated their self-efficacy less strongly following positive, compared to neutral, feedback. Higher self-negativity also associated with sustainment of distress after the speech (Supplementary Results), corroborating the relation of this component to speech-induced negative emotions. We thus focused on self-efficacy update bias in subsequent fMRI analyses.

### Brain encoding of judges’ feedback positivity

**Fig. 4:**
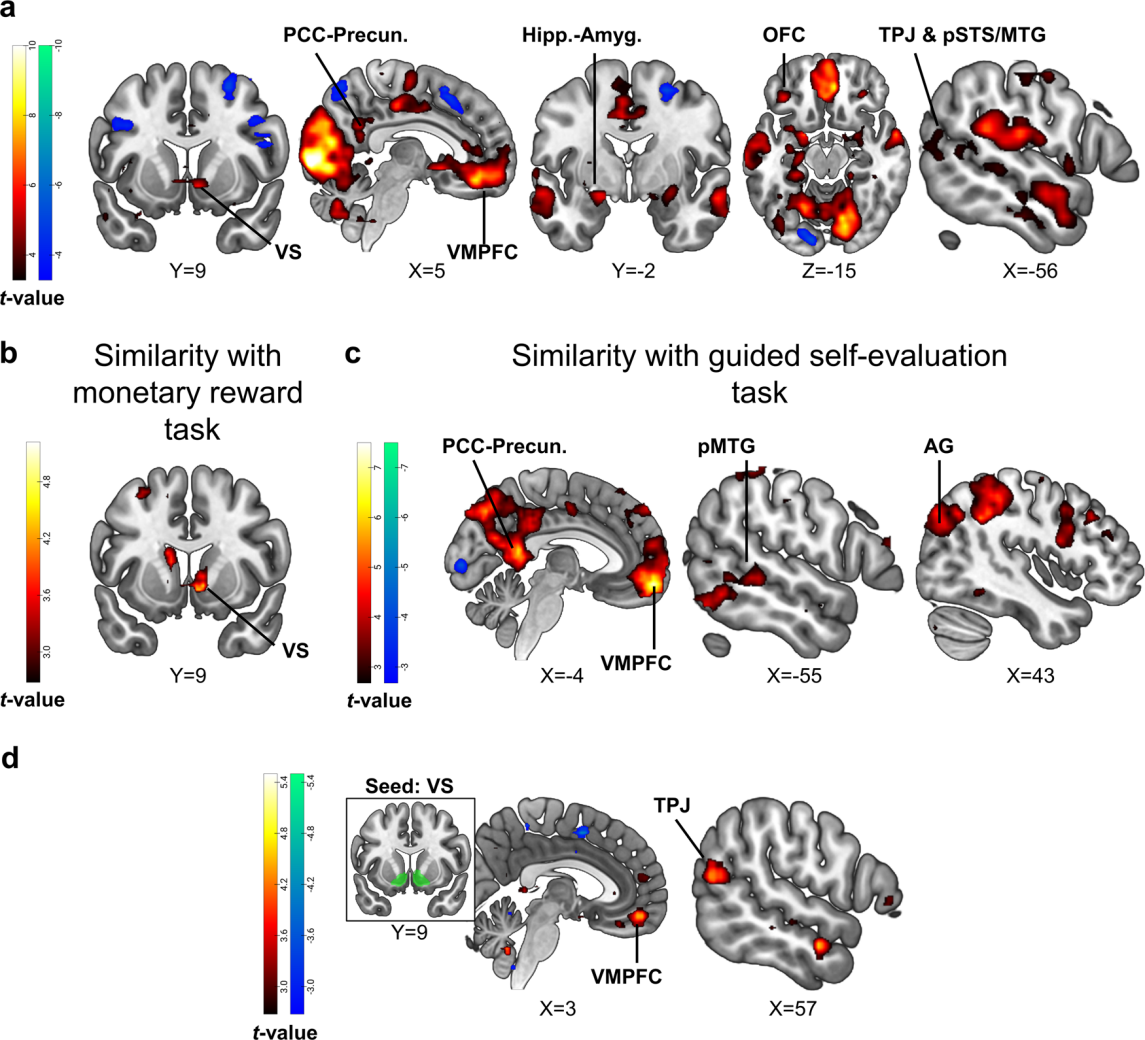
Brain encoding of judges’ feedback positivity. **a** Statistical parametric maps depicting significant activations during the social feedback reception phase. The red-yellow/blue-green color scaling represents positive/negative trial-by-trial correlations of brain activity with feedback scores. Brain maps are thresholded at voxel-level *p* < 0.001 without cluster-extent threshold, for display purposes. **b**, **c** Results from a between-task searchlight similarity analysis. The statistical parametric maps depict similarity between encoding of judges’ feedback positivity and processing of monetary reward (**b**) and guided self-evaluation (**c**). The red-yellow/blue-green color scaling represents the degree of between-task similarity/dissimilarity. **d** VS functional connectivity driven by judges’ feedback positivity. The statistical parametric maps depict results from a whole-brain analysis of bilateral VS functional connectivity during the social feedback reception phase, which was parametrically modulated by judges’ feedback on a trial-by-trial basis. The red-yellow/blue-green color scaling reflects the degree of positive/negative correlation. For display purposes, the brain maps presented in **b**–**d** are thresholded at voxel-level *p* < 0.005, uncorrected, and without cluster-level correction. Brain images are presented in neurological convention (i.e., right is right). VS ventral striatum, VMPFC ventromedial prefrontal cortex, PCC posterior cingulate cortex, precun. precuneus, hipp. hippocampus, amyg. amygdala, OFC orbitofrontal cortex, TPJ temporoparietal junction, pSTS posterior superior temporal sulcus, MTG middle temporal gyrus, AG angular gyrus.

In our fMRI analysis, we first aimed to establish that positive feedback from judges activated our key regions of interest; the VS and VMPFC (known to process reward- and self-evaluation, respectively). Whole-brain analysis revealed that encoding of judges’ feedback positivity correlated positively with brain activity in the VS and VMPFC, as well as in other limbic regions and components of the DMN such as the temporoparietal junction and temporal gyri (Fig. 4a, Supplementary Table 4; voxel-level *p* < 0.001 and cluster-level pFDR < 0.05). Positive feedback also correlated with extensive activation in the occipital cortex, and particularly in the primary visual cortex which is known to respond to reward in humans^73^. Judges’ feedback positivity was anti-correlated with activity in fronto-parietal regions (Fig. 4a, Supplementary Table 4).

To test whether the processing of judges’ feedback positivity involved activating neural correlates of reward and self-evaluation, we next examined results from the searchlight RSA we performed between the social feedback task and each of the auxiliary fMRI tasks. The searchlight RSA between encoding of social feedback positivity (i.e., parametric modulation of feedback by absolute valence>baseline contrast) and monetary reward processing (i.e., winning>losing money contrast; Supplementary Fig. 5) revealed significant similarity in a cluster in the right VS (Fig. 4b, Supplementary Table 5; voxel-level *p* < 0.001 and cluster-level pFDR < 0.05). Searchlight RSA between the social feedback task and the guided self-evaluation task (i.e., attributing traits to oneself>performing a lexical control task on the same traits; Supplementary Fig. 5) revealed significant similarity in clusters in the VMPFC and in additional components of the DMN. The significant similarity between these tasks was also observed in additional components of the default-mode network, including the angular gyri, temporal poles, and middle temporal gyri (Fig. 4c, Supplementary Table 5; voxel-level *p* < 0.001 and cluster-level pFDR < 0.05).

Lastly, we examined whether VS connectivity altered during feedback reception as a function of judges’ feedback positivity. We expected that VS-VMPFC connectivity would reflect the assignment of reward value to oneself. In line with our hypothesis, we found that VS functional connectivity with areas in the VMPFC (and the right temporoparietal junction; TPJ), correlated positively with the encoding of judges’ feedback positivity (Fig. 4d, Supplementary Table 6; voxel-level *p* < 0.001 and cluster-level pFDR < 0.05).

Taken together, these results suggest that positive feedback from judges evoked reward- and self-evaluative-related brain activity in the VS and in the VMPFC, respectively; and further enhanced the functional coupling between these striatal and cortical systems.

### Association of self-efficacy update bias with brain encoding of judges’ feedback positivity

**Fig. 5:**
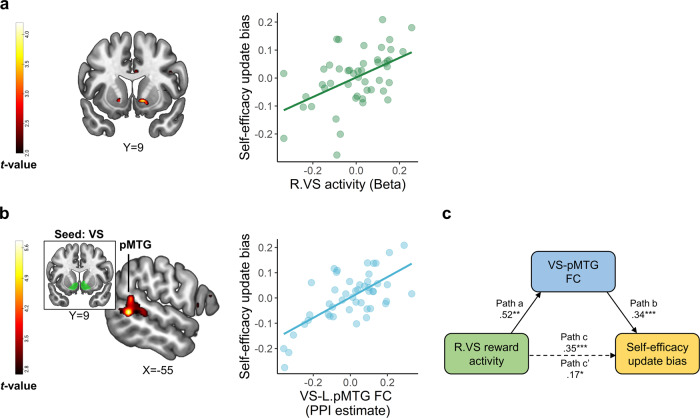
Modulation of ventral striatum activity and connectivity by the positivity of social feedback associates with self-efficacy update bias. **a** Left panel - a statistical parametric map depicting the covariance of brain encoding of judges’ feedback positivity with self-efficacy update bias in the VS. A significant correlation was evident in the right VS (SVC FWE < 0.05). The map is presented at an uncorrected statistical threshold of voxel-level *p* < 0.025, for display purposes. Right panel—to visualize the correlation in the VS, we extracted the mean activity (beta weights) from a 4 mm sphere centered around the coordinates of peak covariance in the VS, and plotted these values against indices of self-efficacy update bias. **b** Whole-brain regression analysis, testing the correlation of self-efficacy update bias with VS functional connectivity reflecting positive social feedback. The scatterplot on the right illustrates the significant correlation in the pMTG cluster (similarly to **a**). The presented brain map is thresholded at voxel-level *p* < 0.005, uncorrected, for display purposes. The scatterplots in panels **a**–**b** by no means present a test result from an independent ROI, and the line denotes a linear trend. **c** Exploratory mediation analysis. Right VS-related increments in self-efficacy update bias were mediated by VS-left pMTG functional connectivity during encoding of judges’ feedback positivity. Asterisks denote significance as follows: ***p < 0.001; **p < 0.005; *p < 0.05. VS ventral striatum, pMTG posterior middle temporal gyrus, R right, L left, FC functional connectivity, PPI psychophysiological interactions. All brain images are presented in neurological convention (i.e., right is right).

We next examined how the encoding of judges’ feedback positivity in the bilateral VS and VMPFC related to self-efficacy update bias. In line with our hypothesis, we found that a stronger response to judges’ feedback positivity in the right VS corresponded with more positive self-efficacy update bias (Fig. 5a; 8 voxels at *x* = 14, *y* = 10, *z* = −7, peak *t*-value = 4.2; voxel-level *p* < 0.001 and small-volume corrected family-wise error (SVC FWE) *p* < 0.05). Self-efficacy update bias also correlated with left VS response, albeit at an uncorrected statistical threshold. This suggests that encoding of judges’ feedback positivity in the VS may contribute to positively biased updating of one’s sense of self-efficacy with regards to future performance. Note that this brain-behavior correlation remained significant also when controlling for the expected feedback score on each trial (Supplementary Results, Supplementary Fig. 6). No other associations between self-efficacy update bias and feedback-related brain activity were found within the inspected ROIs, nor in a whole-brain exploratory analysis. In addition, in a complementary analysis we found that activity in the right VS also negatively encoded pre-speech (aka pre-feedback) self-efficacy ratings during feedback reception (Supplementary Results, Supplementary Fig. 7; this was true also for post-speech self-evaluation ratings). This corroborates the possible involvement of right VS in updating self-efficacy. Lastly, note that exploratory analysis at the ROI and whole-brain level did not reveal any significant neural correlates of individual differences in self-evaluation update bias.

We next examined if VS-VMPFC connectivity reflecting judges’ feedback positivity was strengthened among participants who demonstrated more positive updates of self-efficacy. Contrary to our hypothesis, we did not find significant covariance of self-efficacy update bias with feedback-driven connectivity of the VS to VMPFC. Yet, exploratory analysis at the whole-brain level showed that positive self-efficacy update bias associated with feedback-driven functional coupling between the VS and a cluster in the left posterior middle temporal gyrus (pMTG; Fig. 5b; 181 voxels at *x* = −56, *y* = −50, *z* = 2, peak *t*-value = 5.76; voxel-level *p* < 0.001 and cluster-level pFDR < 0.05).

Thus, self-efficacy update bias was associated with both VS activity and its connectivity to the pMTG, a cortical region that is implicated in comprehending what others think of us^74^. These results suggest that both the VS initial response to rewarding social feedback, and its relay of reward value information to a cortical region potentially involved in the cognitive processing of feedback, may concurrently shape positively biased processing of feedback. This motivated us to test an exploratory mediation model, wherein the positive association between right VS activity and self-efficacy update bias was mediated by the VS-left pMTG functional connectivity. We extracted the peak activity and connectivity values from 4 mm spheres that were centered around coordinates in the VS and pMTG wherein peak significant covariance with self-efficacy update bias was evident. This analysis revealed that higher VS-pMTG connectivity encoding judges’ feedback positivity significantly mediated VS activity-related increments in self-efficacy update bias (bootstrapping mediation analysis; indirect effect of VS-left pMTG functional connectivity: *B* = 0.18, *p* < 0.001, 95% CI: [0.05, 0.35]; Fig. 5c).

## Discussion

In this study, we sought to unveil the psychological and neural mechanisms underlying individual differences in updating performance-related self-beliefs based on positive social feedback. We found that people’s view of their ability to perform on a future speech task (i.e., self-efficacy), was associated with diminished positively biased updating in individuals who rated highly on “self-negativity”—a psychopathological dimension reflecting symptoms of anxiety, depression, and low self-esteem. At the neural level, we found that a less positive self-efficacy update bias was associated with diminished activation in the VS, as well as with weaker connectivity of VS with pMTG, in response to positive social feedback.

The negative association between trait self-negativity and self-efficacy update bias, accords with findings from previous studies of similar public speaking tasks. These studies reported on diminished positively biased processing of social feedback among individuals with symptoms of anxiety, social anxiety and depression^4,16,44^. However, in those studies, positive bias manifested in evaluations of a previously performed speech, whereas we show that a positive update of self-assessments regarding a future event associated with symptoms of psychopathology. Updates regarding one’s self-efficacy to cope with a future speech are arguably more challenging and have greater clinical importance than the retrospective revaluation of a single previous performance since they require a change in one’s beliefs about their characteristic performance and abilities. However, note that the current study is limited in terms of distinguishing between the neural mechanisms of self-beliefs about a past vs. future performance (i.e., self-evaluation and self-efficacy). Self-evaluation and self-efficacy assessments may differ depending on the cognitive and affective components that they involve. Evaluating a past performance may involve processes such as recollecting different aspects of a performance, and perhaps appraising this information and the affective reactions it evokes in relation to longstanding self-beliefs. Assessing one’s self-efficacy to complete an upcoming task might involve the latter processes to some degree, but also their integration with cognitive and motivational processes that uniquely compose self-efficacy. These include visualizing future successful scenarios via goal setting and action planning, and anticipating the outcomes of prospective actions^2^. To better characterize overlapping vs. distinct neural correlates of updating self-evaluation vs. self-efficacy, future studies could measure brain activity differentially during each of these assessments. Nevertheless, note that we attempted to differentiate between the neural correlates of self-evaluation and self-efficacy by examining brain activity encoding the pre-level of their corresponding ratings during feedback reception (Supplementary Results).

Our findings also join recent indications that depressed and anxious individuals are less likely to adjust their beliefs about prospects of facing adverse life events more strongly in accordance with positive rather than negative evidence^75,76^. However, these studies focus on beliefs regarding the likelihood of encountering positive and negative outcomes over which the individual has little control (e.g., suffering from a disease). In contrast, self-efficacy updates such as those measured here capture modification of beliefs regarding the ability to cope with tasks wherein individuals may alter their behavior in order to achieve desired outcomes^18,29^. Updating such beliefs is fundamental for everyday performance situations. Taken together, both the latter research line and our results highlight the need to assess and delineate neuropsychological mechanisms that are implicated in preparing for future events, which may be one of the main challenges of pathological conditions. With regards to the association of self-efficacy updating with psychopathological symptoms, note that we did not find the relation between positive update bias and social anxiety which was stated in our pre-registered hypothesis (see “Methods”). This study did not include clinically diagnosed social anxiety disorder patients, which may exhibit stronger negative bias^4^. Furthermore, the lack of actual social interaction during feedback reception can mitigate socially anxious responses to it^77,78^. Further research is needed to clarify how threatening social cues might influence the processing of positive social feedback in individuals with high social anxiety.

While self-efficacy is key for invigorating motivational processes that lead to personal and professional success (e.g., effort)^2^, it is yet debatable whether self-efficacy beliefs are antecedents of motivational processes or vice versa. Perspectives arguing that motivational factors may be an antecedent of self-efficacy postulate that self-efficacy ratings (i.e., what people say they can do, a common experimental measurement of self-efficacy) may merely reflect what people are motivated to do^79^. A central line of evidence supporting this claim shows that self-efficacy assessments regarding a target behavior may be increased by altering the expected outcomes (e.g., by offering monetary incentives^80^). The latter issue raises the concern that self-efficacy ratings in the current experiment were affected by the monetary prizes we offered in order to encourage participants to complete the speech. However, monetary incentives are less likely to affect self-efficacy rating for behaviors that require specialized skills (e.g., sports performance, and perhaps public speaking), as opposed to behaviors that involve self-regulatory behaviors (e.g., smoking cessation)^80^. Second, the monetary prizes we offered were relatively small (the maximal prize was ~100$) and we told participants that we would inform the winners after the termination of the entire experiment, thereby creating a large psychological distance between the performance and the chance of receiving the monetary prize. Hence, we presume that the effect of the monetary prizes on participants’ motivation to perform the speech task was minimal.

Analysis of the fMRI data revealed that stronger VS activation during encoding of feedback positivity was associated with more positive self-efficacy update bias, and RSA confirmed the activation of a reward-related representation in the VS. Thus, greater engagement of a major reward processing region during feedback on a prior speech, associated with the positive influence of feedback on one’s view of how she or he will perform on a future speech. The link between social reward processing and positive update bias in the VS is consistent with the involvement of the VS in signaling social rewards^13,26,30,81^, guiding reinforcement learning^82^, and optimistic updating of beliefs about future events^27^. In particular, our results accord with a recent finding that individual differences in optimistic updating in an instrumental learning task, correlated most strongly with “low-level” encoding of reward prediction errors in the VS rather than with “high-level” prefrontal activation^66^. Thus, it could be the case that the manifestation of positive bias through self-efficacy updates, are actually cognitive expressions of basic reinforcement learning asymmetry processes occurring at the subcortical level^66^.

Even though VS-VMPFC connectivity was enhanced in response to feedback positivity at the group level, this was not related to individual differences in self-efficacy update bias as expected. However, an exploratory whole-brain analysis revealed that stronger encoding of judges’ feedback positivity via VS-pMTG connectivity, associated with more positive self-efficacy update bias. The pMTG is a central component of the DMN and is frequently activated when people mentalize about the self and others^74,83^. Neuroimaging studies have implicated the pMTG in comparing the value of incoming social feedback against one’s own self-view^13^ and in responding to social praise^84^. Reduced pMTG activity was also found in individuals with low self-esteem when receiving positive social feedback, therefore implicating this region in matching trait self-positivity with feedback^85^. We extend these findings by demonstrating that VS-pMTG relates to how strongly people update their self-efficacy based on feedback. VS-pMTG coupling further mediated the relation between VS response to positive feedback and self-efficacy update bias, thus suggesting that this striatal-cortical pathway may serve to convey positive evidence about the self for the purpose of updating one’s self-efficacy. The association of pMTG with evaluating oneself in a social context was substantiated by the RSA. This analysis showed that encoding judges’ feedback positivity involved activation of multivoxel patterns in several DMN nodes, including the pMTG, that were similar to those activated during self-evaluation that concerned social traits.

Several limitations of this study should be taken into account. While the assignment of feedback to speech performance criteria was random, participants likely differ in how they value different criteria or view them as vital for defining a good performance. Moreover, the assignment of prediction errors was not fully controlled and there were many positive prediction errors in neutral feedback trials, potentially boosting positively biased updating in this condition. Future studies could measure how participants value different performance criteria and manipulate feedback valence and prediction errors independently. Lastly, our study did not include an actual performance of a second speech and objective evaluations of that performance. Estimating a performance that follows social feedback is crucial for characterizing the effect of updating self-efficacy beliefs on one’s actual behavioral change (e.g., as manifested in better performance, reduced anxiety levels etc.).

We identified key neural processes that may promote the preferable integration of positive over neutral social feedback into self-efficacy beliefs. Our findings identify reduced positive bias of this sort as a potential correlate of the psychopathological symptoms of anxiety, depression, and low self-esteem, and join a growing effort to identify maladaptive neurocognitive computations that constitute transdiagnostic psychiatric processes and can guide more precise and personalized therapeutics^86^. Diminished positive updating of self-efficacy may serve as a target for the treatment of clinical disorders involving negative self-views, which to date have mainly attracted therapeutic approaches that focus on mitigating negative emotions or self-related cognitions^87^. Our findings also have implications for disciplines such as education and organizational psychology, since high self-efficacy is essential for performing well in challenging socio-evaluative circumstances that are ever-present within social and occupational realms.

### Supplementary information

Supplementary information

Supplementary Code

## Data Availability

The data that support the findings of this study are available from the corresponding author upon reasonable request.

## Code availability

STAN code for the two hierarchical models we tested is available in the Supplementary Code. Additional code that supports the findings of this study is available from the corresponding author upon reasonable request.
