# Publisher Correction: A corticostriatal pathway mediating self-efficacy enhancement

**DOI:** 10.1038/s44184-022-00007-6

**Published:** 2022-07-25

**Authors:** Ofir Shany, Guy Gurevitch, Gadi Gilam, Netta Dunsky, Shira Reznik Balter, Ayam Greental, Noa Nutkevitch, Eran Eldar, Talma Hendler

**Affiliations:** 1 School of Psychological Sciences, Tel-Aviv University, Tel-Aviv, Israel; 2 Sagol Brain Institute, Tel-Aviv Sourasky Medical Center, Tel-Aviv, Israel; 3 Sackler School of Medicine, Tel-Aviv University, Tel-Aviv, Israel; 4 The Institute of Biomedical and Oral Research, Faculty of Dental Medicine, Hebrew University of Jerusalem, Jerusalem, Israel; 5 Sagol School of Neuroscience, Tel-Aviv University, Tel-Aviv, Israel; 6 Psychology and Cognitive Sciences Departments, Hebrew University of Jerusalem, Jerusalem, Israel

**Keywords:** Personality, Striatum, Reward, Human behaviour

Correction to: *npj Mental Health Research* 10.1038/s44184-022-00006-7, published online 8 July 2022

In this article the ‘Competing Interests’ statement was incorrectly given as ‘The authors declare competing interests’ but should have been ‘The authors declare no competing interests’. The original article has been corrected.

