# Author Correction: A corticostriatal pathway mediating self-efficacy enhancement

**DOI:** 10.1038/s44184-022-00008-5

**Published:** 2022-08-08

**Authors:** Ofir Shany, Guy Gurevitch, Gadi Gilam, Netta Dunsky, Shira Reznik Balter, Ayam Greental, Noa Nutkevitch, Eran Eldar, Talma Hendler

**Affiliations:** 1 School of Psychological Sciences, Tel-Aviv University, Tel-Aviv, Israel; 2 Sagol Brain Institute, Tel-Aviv Sourasky Medical Center, Tel-Aviv, Israel; 3 Sackler School of Medicine, Tel-Aviv University, Tel-Aviv, Israel; 4 The Institute of Biomedical and Oral Research, Faculty of Dental Medicine, Hebrew University of Jerusalem, Jerusalem, Israel; 5 Sagol School of Neuroscience, Tel-Aviv University, Tel-Aviv, Israel; 6 Psychology and Cognitive Sciences Departments, Hebrew University of Jerusalem, Jerusalem, Israel

**Keywords:** Personality, Striatum, Reward, Human behaviour

Correction to: *npj Mental Health Research* 10.1038/s44184-022-00006-7, published online 08 July 2022

The original version of this Article contained an error in the color scaling of Figure 3d. In the original figure, the color scaling adheres to self-negativity values; however, the correct color scaling values should adhere to the self-efficacy update bias. This has now been corrected in the PDF and HTML versions of the Article.

