# Supplementary material for: A corticostriatal pathway mediating self-efficacy enhancement
Source: Npj Ment Health Res. 2022 Jul 8;1:6. doi: 10.1038/s44184-022-00006-7 (PMC10955890; doi:10.1038/s44184-022-00006-7)
Supplement: Supplementary file 1 — Supplementary information [file 44184_2022_6_MOESM1_ESM.pdf]

Supplementary Information

Supplementary Results
Validation of perceived valence of the rating scale
Statistical modelling
Descriptive statistics of positive and neutral components of the update bias indices
Association of self-efficacy update bias with feedback memory
Questionnaire-based components of psychopathology related symptoms
Association of self-negativity with positive vs. neutral components of self-efficacy update bias
Association of self-negativity with speech-induced distress
fMRI results
Brain activity in the auxiliary fMRI tasks
Association of self-efficacy update bias with VS encoding of judges' feedback positivity: complementary analyses
Association of ventral striatum activity with positive vs. neutral components of self-efficacy update bias
Controlling for expected feedback score at the trial-level
Exploration of the neural correlates of self-evaluation and self-efficacy updates
Supplementary Figures
Supplementary Tables
Supplementary Code

Supplementary Results

Validation of perceived valence of the rating scale

In order to characterize the perceived valence of different values on the 11-pt scale that we used for the speech evaluation, we conducted an online experiment on undergraduate psychology students from Tel-Aviv University (n=54; $Mean_{age}$±SD: 22.65 ± 3.16 years, 37 females). Throughout the experiment, participants were first instructed to imagine they have just delivered a public speech, and that professional judges were about to evaluate their performance. Moreover, they were told that they would be evaluated on a 0-10 scale and in comparison to a sample of 100 people that are similar to them in terms of age and education level. Furthermore, they were instructed that on the 0-10 scale, 0 marks the lowest possible score and is thus labeled “much below average”; 5 marks the average performance and is labeled “average” accordingly; and 10 is the maximal score and is therefore labeled “far above average”. Next, the rating scale was presented to participants with numerical indications ranging from 0-10 and the 3 labels mentioned above were positioned in their matching locations. Participants were instructed to mark the range of scores that they believe would be experienced by them as either positive, neutral or negative. Supplementary Figure 1 depicts the association of each score with either positive, neutral or negative valence. Note that more than 50% of the participants associated each of the scores 7-10 with positive valence and scores 5-6 with neutral valence, in accordance with our definition of the positive and neutral feedback conditions. However, the score 4 was associated more often with negative valence than with neutral valence. Regarding this matter, note that the scale used in this experiment differed from the one we actually used in the social feedback experiment in several terms. First, the scale in the online experiment referred to a hypothetical sample of similar others, whereas the scale in the feedback experiment lacked such social reference. Second, the scale in the

online experiment included numerical indications and different labels, which may have influenced participants' ratings negatively (e.g., the score "4" or the label "average", which did not appear in the feedback experiment, may have enhanced the negativity of intermediate scores). Nonetheless, these results confirm that the two conditions differed in terms of their perceived valence.

Statistical modelling

Posterior predictive checks

To validate the accurate performance of the winning model, we implemented posterior predictive checks assessing the correspondence between the actual data and simulated data. To this end, we first generated posterior distributions of data that were simulated based on the estimated model parameters. Thus, for each of the five rating types this procedure resulted in 10,000 posterior samples for each of the 2,000 ratings (i.e. 50 participants x 40 trials). Since our main parameters of interest (Ω_s) reflected the correlation between pairs of ratings for each subject, we computed the Pearson correlation between pre-to-post feedback simulated ratings for each subject on each posterior draw. This was done specifically for correlations between the post-speech self-evaluation and post feedback self-evaluation; as well as for pre-speech self-efficacy and post-feedback self-efficacy. This resulted in a posterior distribution of 10,000 correlation coefficients for each subject, from which we extracted the median. Next, we correlated these simulated correlation coefficients with the actual correlation coefficients describing the coupling between the original ratings across participants, using Pearson correlations (see Supplementary Figure 3).

Descriptive statistics of positive and neutral components of the update bias indices

The update bias indices of interest were computed as the difference between the feedback-rating correlations given positive and neutral feedback. However, as range restriction is known affect the covariance between variables, it might be the case that the covariance on positive vs.

neutral feedback trials varied due to the differential ranges we identified with each of these conditions (i.e. 4-6 for neutral vs. 7-10 for positive). To address this issue, we computed descriptive statistics (mean and standard deviation) for the positive and neutral components for each update bias indices. Descriptive statistic were similar for both the self-evaluation update bias (positive component [M±SD]: 0.43±0.15; neutral component: 0.41±0.16) and the self-efficacy update bias (positive component [M±SD]: 0.39±0.14; neutral component: 0.39±0.18).

<u>Association of self-efficacy update bias with feedback memory</u>

To assess whether self-efficacy update bias was related do differential memory of judges' positive vs. neutral feedback, we performed a control analysis where we tested the correlation between update bias and memory errors. We first computed the Mean Squared Error (MSE) of the difference between the actual and recollected feedback scores under each of the two conditions for each participant. We then subtracted between the memory MSE of each condition, such that $Memory\ MSE_{\Delta} = Memory\ MSE_{pos} - Memory\ MSE_{neu}$. We tested the correlation between $Memory\ MSE_{\Delta}$ and self-efficacy update bias using partial Pearson correlations, where we controlled for individual differences in between-conditions' mean prediction error difference. The correlation between self-efficacy update bias and $Memory\ MSE_{\Delta}$ was not significant (r(47)=-.21, p=.15).

Questionnaire-based components of psychopathology related symptoms

<u>Association of self-negativity with positive vs. neutral components of self-efficacy update bias</u>

One of our main findings was that higher scores on the self-negativity component associated with less positive self-efficacy update bias. The latter was computed as the difference between updates made for positive vs. neutral feedback (see Equation (4) in main text), and this raises the question of whether this correlation was driven more strongly by either positive or neutral

feedback. To test this, we examined the separate correlations of self-negativity with the distinct positive and neutral components of self-efficacy update bias (i.e. $\Omega_{Pos_s(y_3,y_5)}$ and $\Omega_{Neu_s(y_3,y_5)}$, respectively). We used partial Pearson correlations, as we wanted to control for individual differences in the corresponding mean deviance between expected and actual feedback scores (i.e. prediction error) in each of the conditions. None of these correlations were significant (correlation with positive component of self-efficacy update bias: r(47)=.07, p=.65; correlation with neutral component of self-efficacy update bias: r(47)=.15, p=.31). Thus, it seems that the association between self-negativity and self-efficacy update bias was mainly related to the difference between updates made for positive vs. neutral feedback conditions.

Association of self-negativity with speech-induced distress

To test whether self-negativity also associated with changes in subjective distress that were induced by the public speaking task, we correlated the self-negativity scores with the delta in the reported distress between each pair of adjacent ratings. Since we collected five distress ratings, we had four indices of speech-induced distress changes: (1) Difference between distress experienced at baseline (i.e. prior to speech announcement) and just before the speech performance. We assumed that this difference represented anticipatory related distress. (2) Difference between distress experienced just before the speech performance and during the speech performance. We assumed that this difference represented distressful reactivity to the speech. (3) Difference between distress experienced during the speech performance and immediately after its termination. We assumed that this difference represented immediate recovery from the speech-induced distress. (4) Difference between distress experienced immediately after speech termination and subjective distress reported ~35 min later. We assumed that this difference represented long-term sustainment of-, or recovery from-, speech-induced distress. We found that higher self-negativity was

associated with less immediate recovery from speech-induced distress (i.e. difference (3); Spearman correlation: $\rho(48)=.401$, p=.004, pFDR<.05). This suggests that participants who scored higher on this psychopathology related domain, were affected more negatively by the speech performance. None of the correlations between self-negativity and the remaining rating changes were statistically significant (with (1): $\rho(48)=.07$, p=.62; with (2): $\rho(48)=.02$, p=.92; with (4): $\rho(48)=-.15$, p=.29).

fMRI results

<u>Brain activity in the auxiliary fMRI tasks</u>

Prior to conducting the similarity analysis between the social feedback task and the auxiliary tasks, we assured that the latter activated our ROIs. Indeed, VS activation was evident in response to winning vs. losing money in the monetary reward task (Supplementary Figure 5). In the guided self-evaluation task, judging whether traits were descriptive of oneself vs. performing a lexical control task on the same traits activated extensive portions of the default-mode network, including the VMPFC (Supplementary Figure 5).

Association of self-efficacy update bias with VS encoding of judges' feedback positivity: complementary analyses

<u>Association of ventral striatum activity with positive vs. neutral components of self-efficacy update bias</u>

A central result reported in the main text is the association between self-efficacy update bias and VS activity during encoding of judges' positivity. One question surrounding this result is whether this correlation was driven specifically by the delta between update parameters obtained for positive vs. neutral feedback conditions; or whether the positive and neutral components of the update bias (i.e. $\Omega_{Pos_{s(y_3,y_5)}}$ and $\Omega_{Neu_s(y_3,y_5)}$, respectively) contributed separately to this brain-

behavior correlation as well. To inspect this, we computed the covariance between the positive and neutral components of self-efficacy update bias and brain encoding of social feedback positivity in two separate multiple regression analysis, and restricted this analysis to a mask covering the bilateral VS. To control for the potential influence of individual differences in prediction error on both bias parameters and brain activity, we regressed out the mean prediction error during positive or neutral conditions from the corresponding model parameters; and also entered them as between-subject covariates in the relevant regression analysis. To detect significant covariance, we set the statistical threshold at voxel-level $p<.001$ and small-volume corrected family-wise error (SVC FWE) $p<.05$, as we did in the analysis in the main text. This analysis did not reveal any significant covariance of either the positive or neutral components of self-efficacy update bias with VS activity. Nonetheless, we explored the association of the positive or neutral components of self-efficacy update bias with VS activity in a 4mm sphere centered around coordinates in the right VS wherein significant covariance with self-efficacy update bias was observed (x=14, y=10, z=-7). We controlled for individual differences in mean prediction error during positive or neutral conditions here as well, by using partial Pearson correlations. These analyses showed only non-significant trends, suggesting that right VS activity correlated positively with the positive component of self-efficacy update bias ($r(45)=.2$, $p=.18$) and negatively with the neutral component of self-efficacy update bias ($r(45)=-.13$, $p=.39$). Thus, the association we found between self-efficacy update bias and right VS activity encoding judges' positivity was related primarily to the difference between updates made for positive vs. neutral feedback conditions.

Controlling for expected feedback score at the trial-level

A central confound in our experimental design was the deviance between expected and actual social feedback scores (i.e. prediction error) on each trial in the fMRI social feedback task. This

factor varied both across participants and across the positive and neutral conditions. Thus, we controlled for the effect of individual differences in prediction error delta between the positive and neutral conditions, on the different brain functionality indices we examined. Yet, in an additional attempt to control this issue, we modeled the expected feedback scores (i.e. the post-speech self-evaluation scores) at the trial-level, and tested whether brain activations in our ROIs and their association with self-efficacy update bias were affected by this factor. To this end, we executed an additional GLM on the social feedback task fMRI data, which took into account the expected feedback score on each trial. This GLM was similar to the one that is detailed in the Methods section in the main text, except for two main differences. First, the feedback reception phase was modeled with two parametric modulators – one capturing the absolute valence of feedback on each trial, which was our main effect of interest; and another capturing the expected feedback score on each trial. Second, in order to examine the unique effects of the two parametric modulators, their regressors were mean-centered and were not serially orthogonalized[1].

We first questioned if within this design, the absolute valence parametric regressor explained more variance in the VS than the expected score parametric regressor, during the feedback reception phase. Analysis of the contrast between these regressors confirmed that this was the case in the right VS (Supplementary Figure 6, left; voxel-level $p<.001$ SVC $FWE<.05$). Thus, right VS activity upon feedback reception was mainly driven by the absolute valence of feedback scores. Next, we tested if the positive association between self-efficacy update bias and right VS activity during encoding of judges' feedback positivity remained significant also when controlling for the expected score at the trial-level. We repeated the regression analysis that is described in the main text. This analysis affirmed that voxels in the right VS showed significant positive covariance with self-efficacy update bias also when controlling for the expected feedback score on a trial-by-trial

basis (Supplementary Figure 6, right; SVC<.05), but this was evident in fewer voxels (1 voxel at voxel-level $p<.001$ and 5 voxels at $p<.005$; peak t-value=3.6 at x=11, y=7, z=-7).

Exploration of the neural correlates of self-evaluation and self-efficacy updates

A caveat of the current experiment is that we did not record brain activity during the self-evaluation and self-efficacy assessments. Therefor, this study is limited in terms of differentiating between the neural correlates of these self-beliefs and their updating following social feedback (see further discussion in main text). Nonetheless, in an attempt to address this issue, we examined the neural correlates of self-evaluation and self-efficacy during feedback reception. Specifically, we assumed that if feedback is used to update self-efficacy and self-evaluation, then it is possible that the pre-level of both of them is negatively encoded during feedback, while the feedback itself is positively encoded. This is because the prediction errors that drive the updates are ‘feedback minus pre-feedback self-efficacy’ and ‘feedback minus pre-feedback (aka post-speech) self-evaluation’. To test this option, we conducted two additional GLMs. These GLMs were similar to the one used for modelling social feedback in the main text, except that each of them included two parametric regressors during feedback – one for the feedback itself and one for the pre-feedback self-efficacy or self-evaluation ratings, which were modelled on a trial-by-trial basis. In these GLMs the parametric regressors were mean-centered and were not serially orthogonalized.

As mentioned above, we focused this analysis on the *negative* contrast of the parametric regressors of pre-feedback self-evaluation and self-efficacy against baseline. We first examined effects within our key ROIs - the VS and VMPFC. Activity in the right VS negatively encoded both pre-feedback self-evaluation and self-efficacy ratings (Supplementary Figure 7; voxel-level $p\leq.001$ SVC FWE<.05; self-evaluation: 1 voxel at $p\leq.001$ and 6 voxels at $p<.005$, peak t-

value=3.6 at x=7, y=12, z=-7; self-efficacy: 1 voxel at $p \leq .001$ and 6 voxels at $p < .005$, peak t-value=3.16 at x=7, y=10, z=-7). No significant correlation with brain activity was found in the left VS or VMPFC ROIs for both ratings. Next, we explored the neural correlates of self-evaluation and self-efficacy at the whole-brain level. We found that activity in the occipital cortex (lingual gyrus) negatively encoded self-evaluation ratings during feedback (Supplementary Figure 7; voxel-level $p < .001$ and cluster-level $p_{FDR} < .05$; 107 voxels, peak t-value=4.96 at x=9, y=-89, z=-1). We did not find any significant clusters of brain activity that correlated negatively with the self-efficacy ratings during feedback. However, it is noteworthy that at a statistical threshold of voxel-level $p < .001$ and minimal cluster threshold of k=10 (i.e. without cluster-level correction), a cluster of activation in the right hippocampus was evident (Supplementary Figure 7; additional clusters at this threshold included the supplementary motor area and the cuneus). A recent perspective has highlighted the critical role of the hippocampus, a region that is well-known for its role in forming long-term memories, in encoding reward-relevant information that guides future behavior and decision-making[2]. This preliminary result might suggest that updating self-efficacy at the time of feedback may have engaged long-term mnemonic processes, but this option requires further investigation given the lenient statistical threshold of the results. Lastly, it is also noteworthy that although the above-mentioned ROI analyses in the VMPFC were not significant, activity in this area did negatively encode pre-feedback self-evaluation and self-efficacy ratings during feedback at an uncorrected statistical threshold of voxel-level $p < .005$ (Supplementary Figure 7).

Supplementary Figures

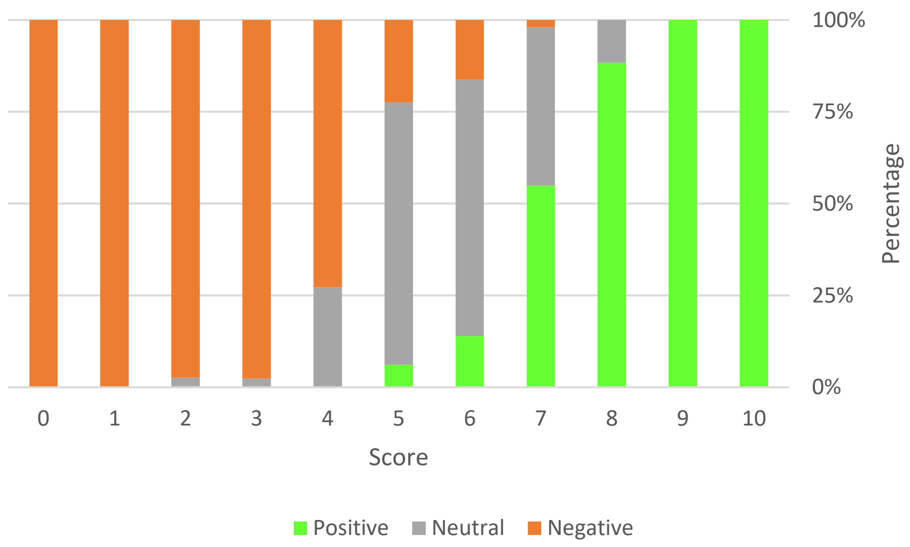

Supplementary Figure 1. Perceived valence of the rating scale values. A stacked bar chart is presented. The chart presents the percentage of participants that associated each possible score on the scale with either positive (light green), neutral (grey) or negative (orange) valence.

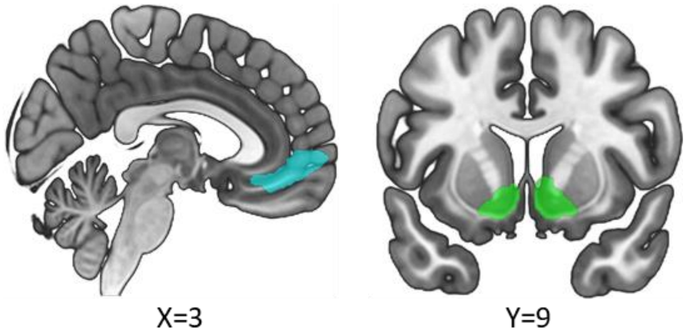

Supplementary Figure 2. Mask used for region-of-interest (ROI) analysis. Left: VMPFC mask, which included regions 41-42 from the Brainnetome atlas[3]. Right: VS mask, which included the Nucleus Accumbens regions from a probabilistic atlas of human subcortical nuclei[4].

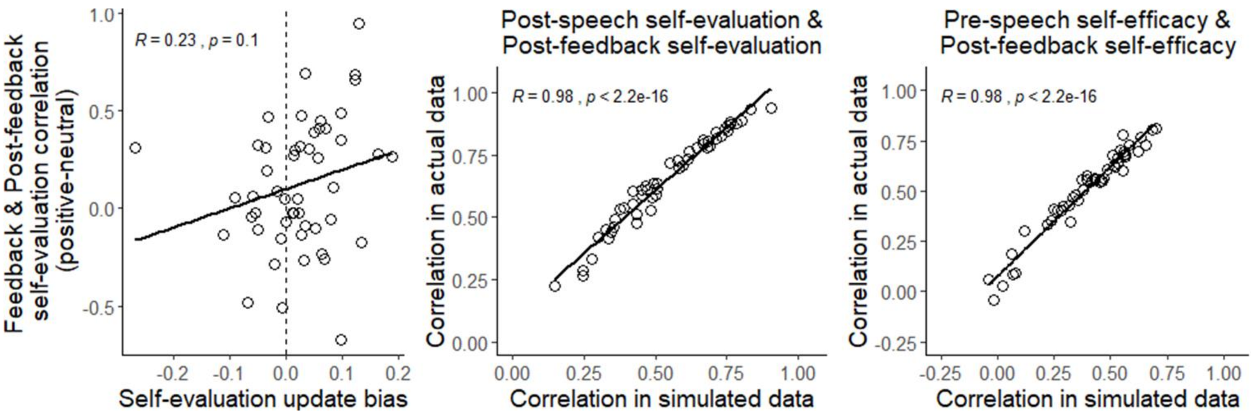

Supplementary Figure 3. Posterior predictive checks of the model. Left panel: a scatterplot depicting the correlation between the self-evaluation update bias indices and the actual correlation differences between the positive and neutral conditions for the feedback and post-feedback self-evaluation pairings. Middle and right panels: scatterplots depicting the linear relation (Pearson correlation) between the actual correlation of pre- and post-feedback pairings and the correlation of the equivalent pair within a simulated dataset. The middle scatterplot depicts the correlation between actual and simulated data for the correlation between post-speech self-evaluation and post-feedback self-evaluation scores. Each point represents correlation coefficients (Pearson's r) for one participants. The right scatterplot shows a similar posterior predictive check for the correlation between pre-speech self-efficacy and post-feedback self-efficacy scores.

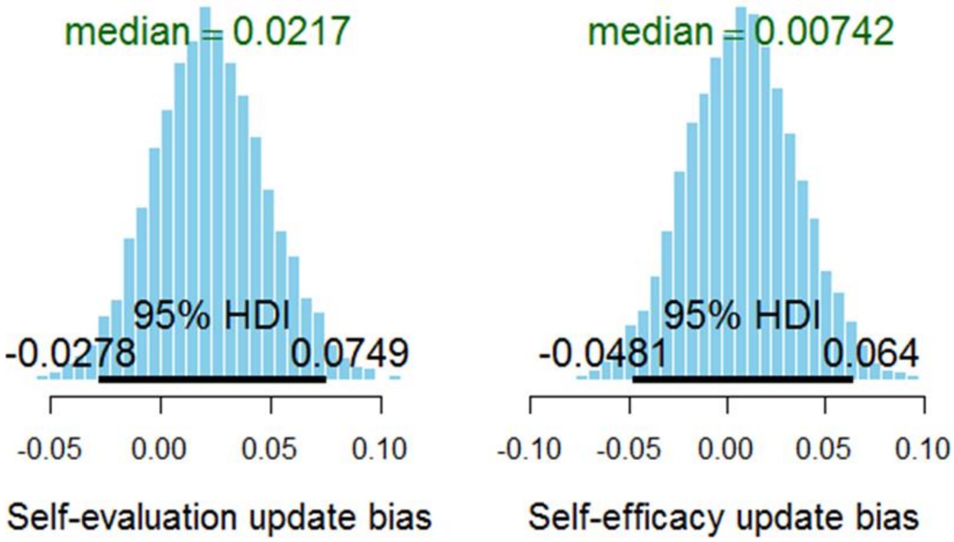

Supplementary Figure 4. Posterior distributions of update bias indices. Posterior distributions of group medians of estimated parameters indicating self-evaluation update bias (left) and self-efficacy update bias (right). The 95% highest density interval (HDI) is marked around the median of the distribution.

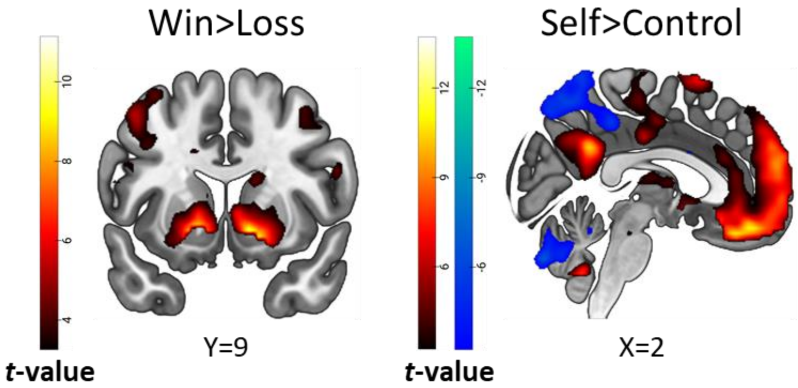

Supplementary Figure 5. Brain activity in the auxiliary fMRI tasks. Left: Statistical parametric map of the winning vs. losing money contrast in the monetary reward task. Right: Statistical parametric map of the self vs. control contrast in the guided self-evaluation task. In both maps, the red-yellow color scaling represents higher activation. The blue-green color scaling in the left map is indicative of greater activation for control vs. self. All brain images are presented in neurological convention (i.e. right is right).

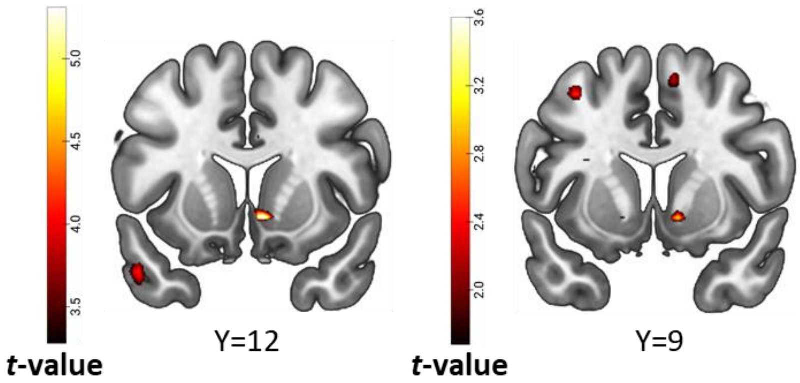

Supplementary Figure 6. Encoding of judges' feedback positivity by ventral striatum activity while controlling for expected feedback scores at the trial-level. Left: The statistical parametric map depicts results from a contrast between two parametric regressors we defined for modelling the response to social feedback reception: one tracking the absolute valence of feedback, and another tracking the expected feedback score. The presented map shows that the absolute valence regressor activated the right VS more strongly than the expected feedback score regressor (voxel-level p<.001, SVC FWE<.05). Right: a statistical parametric map depicting the covariance of brain encoding of judges' feedback positivity with self-efficacy update bias in the right VS, while controlling for the expected feedback scores at the trial level (SVC FWE<.05). The map is presented at an uncorrected statistical threshold of voxel-level p<.05, for display purposes.

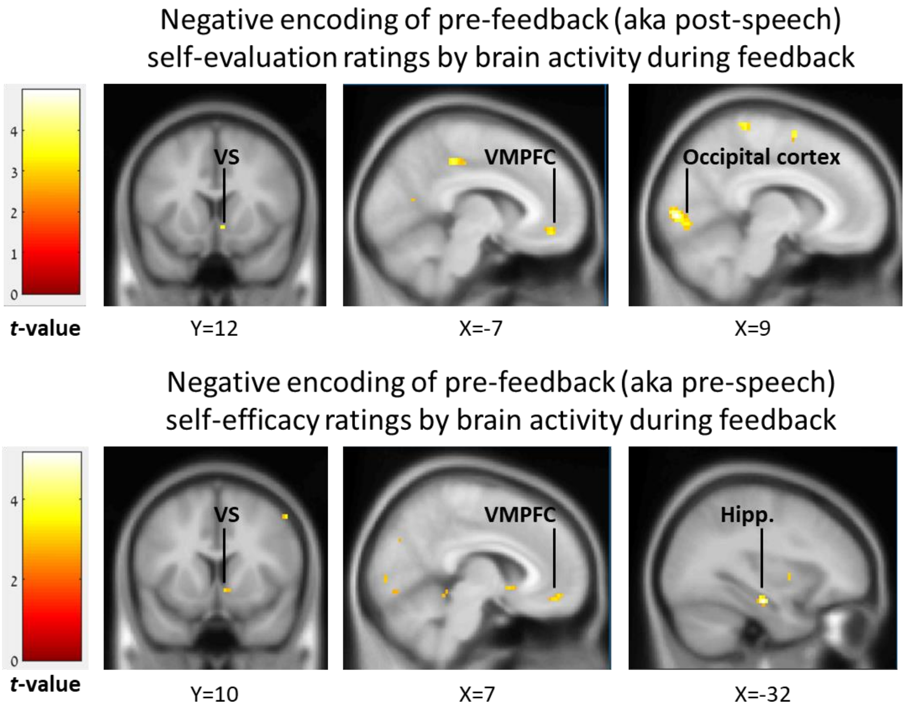

Supplementary Figure 7. Brain encoding of self-evaluation and self-efficacy during feedback. The statistical parametric maps depict significant negative correlation of brain activity with pre-feedback self-evaluation (aka post-speech self-evaluation; upper panel) and self-efficacy ratings (aka pre-speech self-efficacy; lower panel). Both rating types were modelled using a parametric regressor that was aligned with the feedback reception phase, and was modulated by ratings on a trial-by tiral basis. The statistical maps present the contrast of each of these parametric regressors against baseline. The presented brain maps are thresholded at voxel-level $p<.005$ without cluster-extent threshold, for display purposes, and are in neurological convention (right is right). Abbreviations: ventromedial prefrontal cortex (VMPFC); hippocampus (hipp.); ventral striatum (VS).

Supplementary Tables

Feedback item		Reverse coded (1=Yes)
**English**	**Hebrew**	
talked fluently	דיברתי שוטף	0
spoke at a good pace	דיברתי בקצב טוב	0
was authentic	הייתי אותנטי	0
was intelligent	הייתי אינטליגנטי	0
was creative	הייתי יצירתי	0
was charismatic	הייתי כריזמטי	0
was cordial	הייתי לבבי	0
was approachable	הייתי נגיש	0
was understood	הייתי מובן	0
was interesting	הייתי מעניין	0
was convincing	הייתי משכנע	0
was pleasant	הייתי נעים	0
was consistent	הייתי עקבי	0
was eloquent	הייתי רהוט	0
was witty	הייתי שנון	0
was communicative	הייתי תקשורתי	0
was knowledgeable	הפגנתי בקיאות	0
coped with the topic	התמודדתי עם הנושא	0
made eye contact	יצרתי קשר עין	0
spoke clearly	דיברתי ברור	0
seemed energetic	נראיתי אנרגטי	0
seemed natural	נראיתי טבעי	0
seemed prepared	נראיתי מוכן	0
looked professional	נראיתי מקצועי	0
seemed relaxed	נראיתי נינוח	0
was fascinating	הייתי מרתק	0
demonstrated confidence	שידרתי בטחון	0
sounded organized	נשמעתי מאורגן	0
was reasoned	הייתי מנומק	0
spoke monotonically	דיברתי מונוטוני	1
was arrogant	הייתי יהיר	1
was predicted	הייתי צפוי	1
seemed worried	נראיתי מודאג	1
voice trembled	הקול שלי רעד	1
seemed embarrassed	נראיתי נבוך	1
sounded confused	נשמעתי מבולבל	1
was repetitive	הייתי חזרתי	1
was estranged	הייתי מנוכר	1
looked tense	נראיתי מתוח	1
sounded hesitative	נשמעתי מהוסס	1

Supplementary Table 1. Evaluation criteria of speech performance. The last column indicates whether the item was reverse coded in the analysis.

Model	ELPD K-fold	*SE* ELPD K-fold	ELPD K-fold Diff	*SE* Diff
1: Positive vs. neutral feedback	-21853.7	96.859	0	0
2: Positive vs. negative prediction error	-21889.7	96.318	-36.062	14.203

Supplementary Table 2. Model comparison results. The far left column denotes the model name. The second column specifies the summed expected log pointwise predictive density (ELPD; a measure of predictive accuracy) per model based on the K-fold cross-validation, and the third column presents the standard errors of the ELPD. The fourth column shows the difference in ELPD between models. Model 1 has higher predictive density than model *2* (i.e. its ELPD value is higher in 36.062 than that of model 3), thus indicating better performance. The standard error of the between-models differences in ELPD appears in the fifth column. Note that a difference in ELPD (fourth column) that was at least twice the size of the SE of the estimated difference (fifth column), indicates better performance of the model.

Questionnaire	Loadings		
	**Component 1: Social anxiety**	**Component 2: Self-negativity**	**Component 3: Reward sensitivity**
Self-esteem (RSE)	-.167	-.853	-.028
Trait anxiety (STAI-T)	.288	.895	.011
Depression (BDI)	.277	.773	-.015
Neuroticism (NEO-N)	.265	.869	.013
Disqualifying positive social outcomes - self subscale (DPSOS)	.653	.524	-.027
Disqualifying positive social outcomes - other subscale (DPSOS)	.801	.273	.086
Fear of negative evaluation (BFNE)	.892	.346	.033
Fear of positive evaluation (FPES)	.679	.265	-.434
Social anxiety (LSAS)	.924	.134	-.066
Punishment sensitivity (SPSRQ)	.820	.398	.035
Social phobia (SPIN)	.905	.164	-.060
Reward sensitivity (SPSRQ)	-.001	.070	.968

Supplementary Table 3. Principal Components Analysis (PCA) results. The table presents the loadings of all questionnaires that were submitted to the PCA on the three resulting components whose eigenvalue was greater than 1. Note that component 3 was discarded from the analysis. Abbreviations: Rosenberg’s Self-esteem (RSE); State-Trait Anxiety Inventory - Trait (STAI-T); Beck Depression Inventory (BDI); Neuroticism-Extroversion-Openness – Neuroticism (NEO-N); Disqualification of Positive Social Outcomes Scale (DPSOS); Brief Fear of Negative Evaluation scale (BFNE); Fear of Positive Evaluation Scale (FPES); Liebowitz Social Anxiety Scale (LSAS); Sensitivity to Reward and Punishment Questionnaire (SPSRQ); Social Phobia Inventory (SPIN).

Brain regions showing a positive correlation with judges' feedback positivity on a trial-by-trial basis (parametric modulation)

Brain region	L/R	k	Peak t-value	Peak p-value	Cluster size pFDR	MNI coordinates		
						x	y	z
Occipital-, Parietal- & Medial-Temporal Lobes, including Lingual Gyrus, Cuneus, Posterior & Middle Cingulate Cortex, Precuneus & Parahippocampal Gyrus	R	5912	14.17	0.00E+00	0.00E+00	11	-71	-10
Medial Frontal Gyrus, including VMPFC, ACC & VS at (X=7, Y=12, Z=-7)	R	1184	8.72	0.00E+00	0.00E+00	7	42	-16
Postcentral Gyrus	L	579	8.03	0.00E+00	0.00E+00	-44	-25	60
Parietal Lobe and Superior Temporal Gyrus, including Rolandic Operculum & Supramarginal Gyrus	L	341	7.29	0.00E+00	0.00E+00	-48	-22	21
Middle Temporal Gyrus	L	313	5.87	0.00E+00	0.00E+00	-58	-9	-13
Middle Occipital Gyrus	L	186	6.05	0.00E+00	0.00E+00	-48	-84	3
Superior Frontal Gyrus	L	173	6.78	0.00E+00	0.00E+00	-14	44	45
Inferior Parietal Lobule	R	167	6.60	0.00E+00	0.00E+00	48	-32	30
Middle Temporal Gyrus	R	108	7.23	0.00E+00	0.00E+00	59	0	-16
Cerebellum (Vermis VIII)	R	62	6.38	0.00E+00	1.40E-05	4	-68	-34
Precentral Gyrus	R	54	5.77	0.00E+00	4.20E-05	39	-20	60
Cerebellum (Crus I)	R	50	6.10	0.00E+00	7.00E-05	48	-71	-34
Cerebellum (Crus II)	R	48	5.19	2.00E-06	8.80E-05	14	-87	-40
Hippocampus	L	43	5.79	0.00E+00	1.79E-04	-28	-25	-13
Postcentral Gyrus	R	39	6.58	0.00E+00	3.22E-04	27	-45	72
Postcentral Gyrus	R	38	5.53	1.00E-06	3.56E-04	32	-43	60
Angular Gyrus	R	37	5.35	1.00E-06	3.96E-04	48	-61	24
Amygdala	L	36	7.22	0.00E+00	4.44E-04	-19	0	-16
Lateral Orbitofrontal Cortex	L	31	5.87	0.00E+00	1.01E-03	-37	33	-16
Amygdala	R	18	5.02	4.00E-06	1.20E-02	25	0	-13
Middle Frontal Gyrus	L	17	4.67	1.30E-05	1.36E-02	-30	21	48
Parahippocampal Gyrus	L	17	5.61	1.00E-06	1.36E-02	-30	5	-19
Superior Frontal Gyrus	L	15	4.93	5.00E-06	1.87E-02	-14	49	30
Medial Frontal Gyrus (DMPFC)	R	15	4.83	7.00E-06	1.87E-02	9	67	9
Hippocampus	L	15	5.12	3.00E-06	1.87E-02	-30	-11	-22
White matter	L	13	6.56	0.00E+00	2.86E-02	-16	-25	63
Angular Gyrus	L	12	4.69	1.20E-05	3.51E-02	-44	-64	27
Superior Frontal Gyrus	R	11	5.22	2.00E-06	3.80E-02	18	37	48
Anterior Cingulate Cortex	R	11	5.15	3.00E-06	3.80E-02	7	33	12
Rolandic Operculum	R	11	5.31	1.00E-06	3.80E-02	52	5	6
Middle Insula	L	11	5.03	4.00E-06	3.80E-02	-41	0	-1
Lateral Orbitofrontal Cortex	R	11	5.42	1.00E-06	3.80E-02	30	35	-16
Posterior Cingulate Cortex	L	10	4.86	7.00E-06	4.63E-02	0	-13	33
Ventral Striatum	L	10	5.08	3.00E-06	4.63E-02	-7	12	-4

Brain regions showing a positive correlation with judges' feedback positivity on a trial-by-trial basis (parametric modulation)

Brain region	L/R	k	Peak t-value	Peak p-value	Cluster size pFDR	MNI coordinates		
						x	y	z

Brain regions showing a negative correlation with judges' feedback positivity on a trial-by-trial basis (parametric modulation)

Brain region	L/R	k	Peak t-value	Peak p-value	Cluster size pFDR	MNI coordinates		
						x	y	z
Precuneus	R	705	-7.59	0.00E+00	0.00E+00	11	-66	60
Occipital Lobe	L	238	-8.74	0.00E+00	0.00E+00	-12	-94	-1
Inferior Parietal Lobule	L	232	-6.03	0.00E+00	0.00E+00	-46	-50	51
Angular Gyrus	R	182	-6.49	0.00E+00	1.00E-06	39	-75	30
Supplementary Motor Area	L	159	-6.14	0.00E+00	3.00E-06	-7	21	51
Superior Frontal Gyrus	R	132	-5.80	0.00E+00	1.20E-05	25	0	57
Middle Frontal Gyrus	R	107	-4.37	3.40E-05	5.50E-05	41	12	36
Precentral Gyrus	L	59	-4.51	2.20E-05	1.85E-03	-39	10	33
Middle Frontal Gyrus	L	46	-4.56	1.80E-05	5.19E-03	-51	28	27
Middle Frontal Gyrus	R	37	-3.97	1.22E-04	1.10E-02	48	37	21
Inferior Frontal Gyrus	L	26	-4.26	4.80E-05	3.18E-02	-48	19	6
Precuneus	L	25	-4.23	5.40E-05	3.26E-02	-7	-73	48
Inferior Frontal Gyrus	R	21	-4.74	1.00E-05	4.79E-02	50	7	21

Supplementary Table 4. Modulation of brain activation by judges' feedback positivity. The table presents all clusters arising from a whole-brain regression analysis with trial-by-trial absolute feedback valence values as a parametric modulator during the feedback reception phase. The upper/lower parts of the table present regions showing positive/negative correlation with feedback valence. Note that in contrast to the activation map that is presented in the main text (Fig. 4A) that was thresholded at a at voxel-level p<.001 and cluster-level pFDR<.05, here the upper table (i.e. positive correlation with feedback valence) presents results thresholded at voxel-level p<.00005 and cluster-level pFDR<.05. This threshold was set in order to achieve a better distinction between the activated anatomical regions, which could be more informative. The statistical threshold of the lower table (i.e. negative correlation with feedback valence) remained at voxel-level p<.001 and cluster-level pFDR<.05. Coordinates of peak activity are given in MNI space with their peak t-values and corresponding p-values. Anatomical locations were determined using Talairach Daemon. Abbreviations: Peak in Left/Right hemisphere (L/R); dorsomedial prefrontal cortex (DMPFC); ventromedial prefrontal cortex (VMPFC); ventral striatum (VS); anterior cingulate cortex (ACC).

Similarity between social feedback task (positive correlation with judges' feedback positivity> baseline contrast) and monetary reward task (win>loss contrast)

Brain region	L/R	k	Peak t-value	Peak p-value	Cluster size pFDR	MNI coordinates		
						x	y	z
Medial Frontal Gyrus, including DMPFC & ACC	L	117	4.44	3.00E-05	4.39E-04	-3	51	15
Occipital Lobe	L	61	4.39	3.50E-05	1.27E-02	-37	-100	-1
Ventral Striatum	R	41	4.72	1.20E-05	4.79E-02	7	12	-7

Similarity between social feedback task (positive correlation with judges' feedback positivity> baseline contrast) and guided self-evaluation task (self>control contrast)

Brain region	L/R	k	Peak t-value	Peak p-value	Cluster size pFDR	MNI coordinates		
						x	y	z
Precuneus	R	5377	6.82	0.00E+00	0.00E+00	7	-73	45
Medial Frontal Gyrus (VMPFC)	L	823	7.50	0.00E+00	0.00E+00	-5	58	-13
Middle Temporal Gyrus (anterior)	R	723	5.83	0.00E+00	0.00E+00	62	-2	-10
Superior Frontal Gyrus	L	556	5.57	1.00E-06	0.00E+00	-14	39	42
Medial Frontal Gyrus	R	393	6.31	0.00E+00	0.00E+00	30	5	57
Inferior/Middle Temporal Gyrus	L	254	4.91	6.00E-06	0.00E+00	-55	-57	-10
Middle Frontal Gyrus	L	249	4.55	1.90E-05	0.00E+00	-41	19	27
Lingual Gyrus	R	161	5.06	3.00E-06	5.00E-06	16	-75	-16
Cerebellum (Crus I)	R	145	5.39	1.00E-06	1.10E-05	36	-80	-31
Middle Temporal Gyrus	L	134	4.48	2.40E-05	2.00E-05	-64	-13	-16
Middle Temporal Gyrus	R	109	4.49	2.30E-05	9.30E-05	59	-55	-7
Inferior Frontal Gyrus	L	48	4.86	7.00E-06	9.66E-03	-37	35	-19
Postcentral Gyrus	L	42	4.59	1.60E-05	1.55E-02	-41	-20	42
Supramarginal Gyrus	R	41	4.46	2.50E-05	1.58E-02	59	-18	21

Dissimilarity between social feedback task (positive correlation with judges' feedback positivity>baseline contrast) and guided self-evaluation task (self>control contrast)

Brain region	L/R	k	Peak t-value	Peak p-value	Cluster size pFDR	MNI coordinates		
						x	y	z
Lingual Gyrus	L	281	-6.66	0.00E+00	0.00E+00	-14	-87	-13

Supplementary Table 5. Results from a representational similarity searchlight analysis between encoding of judges' feedback positivity and general evaluative processes. The table presents results from a searchlight RSA testing the similarity of brain activity patterns measured during encoding of judges' feedback positivity in the social feedback task with: brain activity evoked by winning vs. losing money in the monetary reward task (upper table); and performing a self vs. control task in the guided self-evaluation task (middle table – similarity; bottom table – dissimilarity). Statistical threshold was set at a voxel-level $p<.001$ and cluster-level $pFDR<.05$. Coordinates of peak activity are given in MNI space with their peak t-values and corresponding p-values. Anatomical locations were determined using Talairach Daemon. Abbreviations: Peak in Left/Right hemisphere (L/R); dorsomedial prefrontal cortex (DMPFC); ventromedial prefrontal cortex (VMPFC); anterior cingulate cortex (ACC).

Brain regions whose functional coupling to the ventral striatum correlated positively with judges' feedback positivity on a trial-by-trial basis (parametric modulation)

Brain region	L/R	k	Peak t-value	Peak p-value	Cluster size pFDR	MNI coordinates		
						x	y	z
Medial Frontal Gyrus (VMPFC)	R	147	4.60	1.60E-05	1.52E-03	2	48	-16
Superior Temporal Gyrus (TPJ)	R	146	4.30	4.20E-05	1.52E-03	62	-54	16

Brain regions whose functional coupling to the ventral striatum correlated negatively with judges' feedback positivity on a trial-by-trial basis (parametric modulation)

Brain region	L/R	k	Peak t-value	Peak p-value	Cluster size pFDR	MNI coordinates		
						x	y	z
Postcentral Gyrus	L	79	-4.90	6.00E-06	5.00E-02	-52	-22	24
Cerebellum	R	75	-4.88	6.00E-06	5.00E-02	18	-64	-52
Lingual Gyrus	R	68	-4.38	3.30E-05	5.00E-02	12	-82	-16
Inferior Parietal Lobule	R	64	-5.41	1.00E-06	5.00E-02	44	-50	52

Supplementary Table 6. Modulation of ventral striatum functional connectivity by judges' feedback positivity. The table presents all clusters arising from a whole-brain regression analysis testing the functional connectivity of the ventral striatum (VS) with the rest of the brain during the feedback reception phase, which was modelled with a trial-by-trial parametric modulator based on the absolute feedback valence values. The upper/lower parts of the table present regions whose connectivity to the VS showed positive/negative correlation with feedback valence. Statistical threshold was set at a voxel-level p<.001 and cluster-level pFDR<.05. Coordinates of peak activity are given in MNI space with their peak t-values and corresponding p-values. Anatomical locations were determined using Talairach Daemon. Abbreviations: Peak in Left/Right hemisphere (L/R); ventromedial prefrontal cortex (VMPFC); temporoparietal junction (TPJ).

Supplementary Code

STAN code for the two models is attached. Explanation about the choice of hyperpriors that were placed on subject- and group-level parameters are presented as well.

References

1. Mumford, J. A., Poline, J.-B. & Poldrack, R. A. Orthogonalization of Regressors in fMRI Models. *PLoS ONE* **10**, e0126255 (2015).
2. Biderman, N., Bakkour, A. & Shohamy, D. What Are Memories For? The Hippocampus Bridges Past Experience with Future Decisions. *Trends in Cognitive Sciences* **24**, 542–556 (2020).
3. Fan, L. *et al.* The Human Brainnetome Atlas: A New Brain Atlas Based on Connectional Architecture. *Cereb. Cortex* **26**, 3508–3526 (2016).
4. Pauli, W. M., Nili, A. N. & Tyszka, J. M. A high-resolution probabilistic in vivo atlas of human subcortical brain nuclei. *Sci Data* **5**, 180063 (2018).
