# Supplementary material for: A corticostriatal pathway mediating self-efficacy enhancement
Source: Npj Ment Health Res. 2022 Jul 8;1:6. doi: 10.1038/s44184-022-00006-7 (PMC10955890; doi:10.1038/s44184-022-00006-7)
Supplement: Supplementary file 2 — Supplementary Code [file 44184_2022_6_MOESM2_ESM.pdf]

Hierarchical structure of candidate models

Below we specify the hierarchical structure and computations of the two models we constructed. Afterwards we present the STAN code for each model. For all parameters we defined vague hyperpriors, in accordance with STAN's manual and wiki (https://mc-stan.org/docs/2_24/stan-users-guide/multivariate-hierarchical-priors-section.html; https://github.com/stan-dev/stan/wiki/Prior-Choice-Recommendations). Note that the depiction of models below is accurate in principle, but the actual implementation used the Cholesky factors of correlation matrices, in order to enhance computational efficiency and numerical stability of the models (see in code).

Model 1: Differential influence of positive vs. neutral feedback

The model:

$$\left[y_{1:5_{s,q}}\right] \sim \mathcal{N}\left(\left[\mu_{1:5_s}\right], \Sigma_{Pos_s}\right)$$

$$for\ y_{3_{s,q}} \geq 7$$

$$\left[y_{1:5_{s,q}}\right] \sim \mathcal{N}\left(\left[\mu_{1:5_s}\right], \Sigma_{Neu_s}\right)$$

$$for\ y_{3_{s,q}} < 7$$

Where $y_{1:5}$ denote the series of speech assessment scores – namely pre-speech self-efficacy (y_1), post-speech self-evaluation (y_2), the judges' feedback (y_3), post-feedback self-evaluation (y_4) and post-feedback self-efficacy(y_5). The model describes these scores as drawn from a multivariate normal distribution. The μ_s parameters denote the subject-level mean for each score type. The Σ_s parameters are covariance matrices whose diagonal describe the variance of each score, and the off-diagonal elements describe the coupling between each pair of scores. Below we specify the hierarchical structure and computations of the μ_s parameters and of the covariance matrices Σ_{Pos_s} and Σ_{Neu_s}.

Priors for μ_s:

Each subject-level μ_s is sampled with group-level parameters (indexed g hereinafter) as follows:

$$Number\ of\ responses\ on\ scale: N_{resp} = 11$$

$$\sigma_g^2 \sim \mathcal{U}(0.001, N_{resp})$$

$$\mu_g \sim \mathcal{U}(1, N_{resp})$$

$$\mu_s \sim \mathcal{N}(\mu_g, \sigma_g)$$

Note that the mean judges' rating (i.e. y_3) was fixed on the mean value (7.65) and not estimated, since it was known and equal for all participants.

Priors for Σ_s:

The two Σ_s (i.e. Σ_{Pos_s} and Σ_{Neu_s}) were computed by independently sampling two covariance matrices that describe the relation between the different scores – one matrix for positive feedback ($\Sigma_{PosToNeu_s}$) and one for neutral feedback ($\Sigma_{NeuToPos_s}$). Since we thought there might be some degree of dependency between the matrices (for example, participants who are more influenced by positive feedback relative to other participants may be more influenced by neutral feedback as well), we represented this possible dependence by mixing the matrices to a degree that depends on a free parameter P. This parameter receives values ranging between 0.5-1, such that a value of 0.5 leads to identical matrices, whereas a value of 1 leads to completely independent matrices.

The prior for the free parameter $P_{PosToNeu}$ is sampled as follows:

$$P_{PosToNeu} \sim \mathcal{U}(0.5, 1)$$

Σ_{Pos_s} and Σ_{Neu_s} are then defined in the following manner:

$$\Sigma_{Pos_s} = \left(\Sigma_{NeuToPos_s} \times P_{PosToNeu}\right) + \left(\Sigma_{PosToNeu_s} \times \left(1 - P_{PosToNeu}\right)\right)$$

$$\Sigma_{Neu_s} = \left(\Sigma_{NeuToPos_s} \times \left(1 - P_{PosToNeu}\right)\right) + \left(\Sigma_{PosToNeu_s} \times P_{PosToNeu}\right)$$

In the equation above $\Sigma_{PosToNeu_s}$ and $\Sigma_{NeuToPos_s}$ are the quadratic form of the relevant matrix Ω_s and the diagonal matrix of the vector τ_s as follows:

$$Q = \begin{bmatrix} \tau_1 & 0 & 0 \\ 0 & \ddots & \vdots \\ 0 & \dots & \tau_k \end{bmatrix} \times \Omega_s \times \begin{bmatrix} \tau_1 & 0 & 0 \\ 0 & \ddots & \vdots \\ 0 & \dots & \tau_k \end{bmatrix}$$

$$\Sigma_{PosToNeu_s} = Q(\Omega_{PosToNeu_s}, \tau_s)$$

$$\Sigma_{NeuToPos_s} = Q(\Omega_{NeuToPos_s}, \tau_s)$$

The priors for the subject-level parameters Ω_s and τ_s are sampled as follows:

Priors for Ω_s:

$$\Omega_{PosToNeu_s} \sim \text{LKJCorr}(1)$$

$$\Omega_{NeuToPos_s} \sim \text{LKJCorr}(1)$$

Priors for τ_s:

$$\mu_\tau \sim \text{Cauchy}(0, 2.5)$$

$$\sigma_\tau \sim \mathcal{U}(0.001, N_{resp})$$

$$\tau_s \sim \Gamma\left(\left(\frac{\mu_\tau}{\sigma_\tau}\right)^2, \frac{\mu_\tau}{\sigma_\tau^2}\right)$$

Model 2: Differential influence of positive vs. negative prediction error

The model:

This model was similar to model 1, apart from its conditioning on prediction error, in the sense that a different covariance matrix was implemented for positive and negative deviations of judges' actual feedback (i.e. y_3) from participants' expectations (i.e. y_2):

$$\left[y_{1:5_{s,q}}\right] \sim \mathcal{N}\left(\left[\mu_{1:5_s}\right], \Sigma_{Pos_s}\right)$$

$$for\ (y_{3_{s,q}} - y_{2_{s,q}}) > 0$$

$$\left[y_{1:5_{s,q}}\right] \sim \mathcal{N}\left(\left[\mu_{1:5_s}\right], \Sigma_{Neg_s}\right)$$

$$for\ (y_{3_{s,q}} - y_{2_{s,q}}) < 0$$

$$\left[y_{1:5_{s,q}}\right] \sim \mathcal{N}\left(\left[\mu_{1:5_s}\right], \Sigma_{Zero_s}\right)$$

$$for\ (y_{3_{s,q}} - y_{2_{s,q}}) = 0$$

Estimation of all model parameters was identical to model 1. The only difference was the computation of the covariance matrix Σ_{Zero_s}, which captured questions in which the feedback scores matched with participants' expectations. Σ_{Zero_s} is computed as an average of the two above-defined matrices:

$$\Sigma_{Zero_s} = (\Sigma_{Pos_s} \times 0.5) + (\Sigma_{Neg_s} \times 0.5)$$

STAN code for model 1:

```
data{
int <lower=1> Nsubj ; // number of participants
int <lower=1> Ntotal ; // number of questions
int <lower=1> nYlevels ; // number of responses on scale (0-10 scale, so 11 responses)
int <lower=1> s[Ntotal] ; // index of subject at a given question
int <lower=1> k ; // num. of predictors
int  yPreA[Ntotal] ; // this is pre-speech self-efficacy
int  yPreFb[Ntotal] ; // this is post-speech self-evaluation
int  yJud[Ntotal] ;  // judges' feedback
int  yPost[Ntotal] ; // this is post-feedback self-evaluation
int  yPreB[Ntotal] ;  // this is post-feedback self-efficacy
}

transformed data {
int omega_corr_postoneg = 1;
int omega_corr_negtopos = 1;
real muJudPosSub = 7.65;
}

parameters {

real <lower=0> mu_tau  ;
real <lower=0.001, upper=nYlevels> sigma_tau ;

vector<lower=0>[k] tau[Nsubj] ;
cholesky_factor_corr[k] Omega_negtopos[Nsubj] ;
cholesky_factor_corr[k] Omega_postoneg[Nsubj] ;
real <lower=0.5,upper=1> postoneg_prop;

vector[Nsubj] muPreASub ;
real <lower=1,upper=nYlevels> muPreAGroup ;
real <lower=0.001,upper=nYlevels> sigma2PreAGroup ;

vector[Nsubj] muPreFbSub ;
real <lower=1,upper=nYlevels> muPreFbGroup ;
real <lower=0.001,upper=nYlevels> sigma2PreFbGroup ;

vector[Nsubj] muPostSub ;
real <lower=1,upper=nYlevels> muPostGroup ;
real <lower=0.001,upper=nYlevels> sigma2PostGroup ;

vector[Nsubj] muPreBSub ;
real <lower=1,upper=nYlevels> muPreBGroup ;
real <lower=0.001,upper=nYlevels> sigma2PreBGroup ;

}
```

```
transformed parameters{

matrix[k, k] quad_pos[Nsubj];
matrix[k, k] quad_neg[Nsubj];

for (i in 1:Nsubj) {
matrix[k, k] quad_negtopos;
matrix[k, k] quad_postoneg;
quad_negtopos = diag_pre_multiply(tau[i,], Omega_negtopos[i,,]);
quad_postoneg = diag_pre_multiply(tau[i,], Omega_postoneg[i,,]);
quad_pos[i,,] = quad_negtopos*postoneg_prop + quad_postoneg*(1-postoneg_prop);
quad_neg[i,,] = quad_negtopos*(1-postoneg_prop) + quad_postoneg*postoneg_prop;
}
}

model {

mu_tau ~ cauchy(0, 2.5);
sigma_tau ~ uniform(0.001, nYlevels);

for (i in 1:Nsubj) {
tau[i,] ~ gamma((mu_tau/sigma_tau)^2, mu_tau/(sigma_tau^2));
Omega_postoneg[i,,] ~ lkj_corr_cholesky(omega_corr_postoneg);
Omega_negtopos[i,,] ~ lkj_corr_cholesky(omega_corr_negtopos);
}

postoneg_prop ~ uniform(0.5,1);

sigma2PreAGroup ~ uniform(0.001, nYlevels);
muPreAGroup ~ uniform(1 , nYlevels);
muPreASub ~ normal(muPreAGroup, sqrt(sigma2PreAGroup)) ;

sigma2PreFbGroup ~ uniform(0.001, nYlevels);
muPreFbGroup ~ uniform(1 , nYlevels);
muPreFbSub ~ normal(muPreFbGroup, sqrt(sigma2PreFbGroup)) ;

sigma2PostGroup ~ uniform(0.001, nYlevels);
muPostGroup ~ uniform(1 , nYlevels);
muPostSub ~ normal(muPostGroup, sqrt(sigma2PostGroup)) ;

sigma2PreBGroup ~ uniform(0.001, nYlevels);
muPreBGroup ~ uniform(1 , nYlevels);
muPreBSub ~ normal(muPreBGroup, sqrt(sigma2PreBGroup)) ;

for (i in 1:Ntotal) {
if (yJud[i]>6) {
[yPreA[i], yPreFb[i], yJud[i], yPost[i], yPreB[i]] ~ multi_normal_cholesky([muPreASub[s[i]],
muPreFbSub[s[i]], muJudPosSub, muPostSub[s[i]], muPreBSub[s[i]]], quad_pos[s[i],,]);
}
```

```
else if (yJud[i]<7) {
[yPreA[i], yPreFb[i], yJud[i], yPost[i], yPreB[i]] ~ multi_normal_cholesky([muPreASub[s[i]],
muPreFbSub[s[i]], muJudPosSub, muPostSub[s[i]], muPreBSub[s[i]]], quad_neg[s[i],,]);
}
}
}

generated quantities {

vector[k] y_new[Ntotal];

vector[k] D_pos[Nsubj];
vector[k] D_neg[Nsubj];

corr_matrix[k] Omega_pos_true[Nsubj];
corr_matrix[k] Omega_neg_true[Nsubj];

// convert covariance matrix to correlation matrix:

for (i in 1:Nsubj) {
D_pos[i] = sqrt(diagonal(multiply_lower_tri_self_transpose(quad_pos[i,,])));
Omega_pos_true[i] = inverse(diag_matrix(D_pos[i])) * multiply_lower_tri_self_transpose(quad_pos[i,,]) *
inverse(diag_matrix(D_pos[i]))';
D_neg[i] = sqrt(diagonal(multiply_lower_tri_self_transpose(quad_neg[i,,])));
Omega_neg_true[i] = inverse(diag_matrix(D_neg[i])) * multiply_lower_tri_self_transpose(quad_neg[i,,]) *
inverse(diag_matrix(D_neg[i]))';
}

// generate simulated data:

for (i in 1: Ntotal) {
if (yJud[i]>6) {
y_new[i,1:k] = multi_normal_cholesky_rng([muPreASub[s[i]],
muPreFbSub[s[i]], muJudPosSub, muPostSub[s[i]], muPreBSub[s[i]]], quad_pos[s[i],,]);
}
if (yJud[i]<7) {
y_new[i,1:k] = multi_normal_cholesky_rng([muPreASub[s[i]],
muPreFbSub[s[i]], muJudPosSub, muPostSub[s[i]], muPreBSub[s[i]]], quad_neg[s[i],,]);
}
}
}
```

<u>STAN code for model 2:</u>

```
data{
int <lower=1> Nsubj ; // number of participants
int <lower=1> Ntotal ; // number of questions
int <lower=1> nYlevels ; // number of responses on scale (0-10 scale, so 11 responses)
int <lower=1> s[Ntotal] ; // index of subject at a given question
int <lower=1> k ; // num. of predictors
int  yPreA[Ntotal] ; // this is pre-speech self-efficacy
int  yPreFb[Ntotal] ; // this is post-speech self-evaluation
int  yJud[Ntotal] ;  // judges' feedback
int  yPost[Ntotal] ; // this is post-feedback self-evaluation
int  yPreB[Ntotal] ;  // this is post-feedback self-efficacy
}

transformed data {
int omega_corr_postoneg = 1;
int omega_corr_negtopos = 1;
real muJudPosSub = 7.65;
}

parameters {

real <lower=0> mu_tau  ;
real <lower=0.001, upper=nYlevels> sigma_tau ;

vector<lower=0>[k] tau[Nsubj] ;
cholesky_factor_corr[k] Omega_negtopos[Nsubj] ;
cholesky_factor_corr[k] Omega_postoneg[Nsubj] ;
real <lower=0.5,upper=1> postoneg_prop;

vector[Nsubj] muPreASub ;
real <lower=1,upper=nYlevels> muPreAGroup ;
real <lower=0.001,upper=nYlevels> sigma2PreAGroup ;
```

```
vector[Nsubj] muPreFbSub ;
real <lower=1,upper=nYlevels> muPreFbGroup ;
real <lower=0.001,upper=nYlevels> sigma2PreFbGroup ;

vector[Nsubj] muPostSub ;
real <lower=1,upper=nYlevels> muPostGroup ;
real <lower=0.001,upper=nYlevels> sigma2PostGroup ;

vector[Nsubj] muPreBSub ;
real <lower=1,upper=nYlevels> muPreBGroup ;
real <lower=0.001,upper=nYlevels> sigma2PreBGroup ;

}


transformed parameters{

matrix[k, k] quad_pos[Nsubj];
matrix[k, k] quad_neg[Nsubj];
matrix[k, k] quad_zero[Nsubj];

for (i in 1:Nsubj) {
matrix[k, k] quad_negtopos;
matrix[k, k] quad_postoneg;
quad_negtopos = diag_pre_multiply(tau[i,], Omega_negtopos[i,,]);
quad_postoneg = diag_pre_multiply(tau[i,], Omega_postoneg[i,,]);
quad_pos[i,,] = quad_negtopos*postoneg_prop + quad_postoneg*(1-postoneg_prop);
quad_neg[i,,] = quad_negtopos*(1-postoneg_prop) + quad_postoneg*postoneg_prop;
quad_zero[i,,] = quad_pos[i,,]*0.5 + quad_neg[i,,]*0.5;
}
}

model {
```

```
mu_tau ~ cauchy(0, 2.5);
sigma_tau ~ uniform(0.001, nYlevels);

for (i in 1:Nsubj) {
tau[i,] ~ gamma((mu_tau/sigma_tau)^2, mu_tau/(sigma_tau^2));
Omega_postoneg[i,,] ~ lkj_corr_cholesky(omega_corr_postoneg);
Omega_negtopos[i,,] ~ lkj_corr_cholesky(omega_corr_negtopos);
}

postoneg_prop ~ uniform(0.5,1);

sigma2PreAGroup ~ uniform(0.001, nYlevels);
muPreAGroup ~ uniform(1 , nYlevels);
muPreASub ~ normal(muPreAGroup, sqrt(sigma2PreAGroup)) ;

sigma2PreFbGroup ~ uniform(0.001, nYlevels);
muPreFbGroup ~ uniform(1 , nYlevels);
muPreFbSub ~ normal(muPreFbGroup, sqrt(sigma2PreFbGroup)) ;

sigma2PostGroup ~ uniform(0.001, nYlevels);
muPostGroup ~ uniform(1 , nYlevels);
muPostSub ~ normal(muPostGroup, sqrt(sigma2PostGroup)) ;

sigma2PreBGroup ~ uniform(0.001, nYlevels);
muPreBGroup ~ uniform(1 , nYlevels);
muPreBSub ~ normal(muPreBGroup, sqrt(sigma2PreBGroup)) ;

for (i in 1:Ntotal) {

if (yJud[i]-yPreFb[i]>0) {
[yPreA[i], yPreFb[i], yJud[i], yPost[i], yPreB[i]] ~ multi_normal_cholesky([muPreASub[s[i]],
muPreFbSub[s[i]], muJudPosSub, muPostSub[s[i]], muPreBSub[s[i]]], quad_pos[s[i],,]);
}
```

```
else if (yJud[i]-yPreFb[i]<0) {
[yPreA[i], yPreFb[i], yJud[i], yPost[i], yPreB[i]] ~ multi_normal_cholesky([muPreASub[s[i]],
muPreFbSub[s[i]], muJudPosSub, muPostSub[s[i]], muPreBSub[s[i]]], quad_neg[s[i],,]);
}

else if (yJud[i]-yPreFb[i]==0) {
[yPreA[i], yPreFb[i], yJud[i], yPost[i], yPreB[i]] ~ multi_normal_cholesky([muPreASub[s[i]],
muPreFbSub[s[i]], muJudPosSub, muPostSub[s[i]], muPreBSub[s[i]]], quad_zero[s[i],,]);
}
}
}

generated quantities {

vector[k] y_new[Ntotal];

vector[k] D_pos[Nsubj];
vector[k] D_neg[Nsubj];
vector[k] D_zero[Nsubj];

corr_matrix[k] Omega_pos_true[Nsubj];
corr_matrix[k] Omega_neg_true[Nsubj];
corr_matrix[k] Omega_zero_true[Nsubj];

// convert covariance matrix to correlation matrix:

for (i in 1:Nsubj) {
D_pos[i] = sqrt(diagonal(multiply_lower_tri_self_transpose(quad_pos[i,,])));
Omega_pos_true[i] = inverse(diag_matrix(D_pos[i])) * multiply_lower_tri_self_transpose(quad_pos[i,,]) *
inverse(diag_matrix(D_pos[i]))';

D_neg[i] = sqrt(diagonal(multiply_lower_tri_self_transpose(quad_neg[i,,])));
```

```
Omega_neg_true[i] = inverse(diag_matrix(D_neg[i])) * multiply_lower_tri_self_transpose(quad_neg[i,,]) *
inverse(diag_matrix(D_neg[i]))';

D_zero[i] = sqrt(diagonal(multiply_lower_tri_self_transpose(quad_zero[i,,])));
Omega_zero_true[i] = inverse(diag_matrix(D_zero[i])) * multiply_lower_tri_self_transpose(quad_zero[i,,]) *
inverse(diag_matrix(D_zero[i]))';

}

// generate simulated data:

for (i in 1: Ntotal) {

if (yJud[i]-yPreFb[i]>0) {
y_new[i,1:k] = multi_normal_cholesky_rng([muPreASub[s[i]],
muPreFbSub[s[i]], muJudPosSub, muPostSub[s[i]], muPreBSub[s[i]]], quad_pos[s[i],,]);
}

if (yJud[i]-yPreFb[i]<0) {
y_new[i,1:k] = multi_normal_cholesky_rng([muPreASub[s[i]],
muPreFbSub[s[i]], muJudPosSub, muPostSub[s[i]], muPreBSub[s[i]]], quad_neg[s[i],,]);
}

if (yJud[i]-yPreFb[i]==0) {
y_new[i,1:k] = multi_normal_cholesky_rng([muPreASub[s[i]],
muPreFbSub[s[i]], muJudPosSub, muPostSub[s[i]], muPreBSub[s[i]]], quad_zero[s[i],,]);
}
}
}
```
